# Flavones provide resistance to DUX4-induced toxicity via an mTor-independent mechanism

**DOI:** 10.1038/s41419-023-06257-2

**Published:** 2023-11-16

**Authors:** Justin Cohen, Shushu Huang, Katherine E. Koczwara, Kristen T. Woods, Vincent Ho, Keryn G. Woodman, Jack L. Arbiser, Katelyn Daman, Monkol Lek, Charles P. Emerson, Alec M. DeSimone

**Affiliations:** 1 Department of Genetics, Yale School of Medicine, New Haven, CT 06510 USA; 2 Wellstone Muscular Dystrophy Program, Department of Neurology, University of Massachusetts Chan Medical School, Worcester, MA USA; 3 Li Weibo Institute for Rare Disease Research University of Massachusetts Chan Medical School, Worcester, MA USA; 4 United Derm Partners/Metroderm, Atlanta, GA 30322 USA; 5 Present Address: Modalis Therapeutics, Waltham, MA USA

**Keywords:** Diseases, Autophagy, Apoptosis, Cell signalling

## Abstract

Facioscapulohumeral muscular dystrophy (FSHD) is among the most common of the muscular dystrophies, affecting nearly 1 in 8000 individuals, and is a cause of profound disability. Genetically, FSHD is linked to the contraction and/or epigenetic de-repression of the *D4Z4* repeat array on chromosome 4, thereby allowing expression of the *DUX4* gene in skeletal muscle. If the *DUX4* transcript incorporates a stabilizing polyadenylation site the myotoxic DUX4 protein will be synthesized, resulting in muscle wasting. The mechanism of toxicity remains unclear, as many DUX4-induced cytopathologies have been described, however cell death does primarily occur through caspase 3/7-dependent apoptosis. To date, most FSHD therapeutic development has focused on molecular methods targeting *DUX4* expression or the *DUX4* transcript, while therapies targeting processes downstream of DUX4 activity have received less attention. Several studies have demonstrated that inhibition of multiple signal transduction pathways can ameliorate DUX4-induced toxicity, and thus compounds targeting these pathways have the potential to be developed into FSHD therapeutics. To this end, we have screened a group of small molecules curated based on their reported activity in relevant pathways and/or structural relationships with known toxicity-modulating molecules. We have identified a panel of five compounds that function downstream of DUX4 activity to inhibit DUX4-induced toxicity. Unexpectedly, this effect was mediated through an mTor-independent mechanism that preserved expression of ULK1 and correlated with an increase in a marker of active cellular autophagy. This identifies these flavones as compounds of interest for therapeutic development, and potentially identifies the autophagy pathway as a target for therapeutics.

## Introduction

With an estimated prevalence of nearly 1 in 8000 individuals [1] Facioscapulohumeral Muscular Dystrophy (FSHD) is one of the most common muscular dystrophies. Pathology typically begins with weakness in the facial, scapular, and humeral muscles, but progresses to the trunk and lower extremities, resulting in profound disability [2]. FSHD is most often inherited as a dominant Mendelian trait, however the genetic etiology is complex. Disease is associated with a repeat array of 3.3 kb *D4Z4* elements located near the telomere of chromosome 4q [3, 4]. In the unaffected population the array most often contains between approximately 10 and 100 units. The most common form of the disease, FSHD1, results from contraction of the array below ~9 units, which allows epigenetic de-repression of the *DUX4* gene contained within each repeat (reviewed in [2]). Contraction alone does not cause disease however, as it must occur on a chromosome carrying a “permissive” 4qA haplotype, which allows the transcript expressed from the last repeat to incorporate a signal that allows polyadenylation of the mRNA, which stabilizes it and enables DUX4 protein synthesis [5–12]. In the second, less common, form of the disease, FSHD2, *trans*-acting mutations in *SMCHD1*, *DNMT3B*, or *LRIF1* cause epigenetic de-repression of the array, and in the presence of a permissive 4qA allele can result in transcription of the stabilized *DUX4* [5, 13–16]. Oddly, even when all genetic determinants of disease are present, *DUX4* is not uniformly expressed, but instead activation occurs in “bursts” in a small fraction of myonuclei [17–21].

DUX4 is a double homeobox transcription factor with well-characterized target genes [6, 22–25]. It has a biological function in the regulation of zygotic genome activation and the oocyte-to-embryo transition [26–29], and is also expressed strongly in testis [17], but it is normally absent in muscle. Despite much study, a detailed model of how DUX4 causes pathology has not emerged. DUX4 is pro-apoptotic [25] and toxic to muscle in many cellular and animal disease models [30]. Cell death occurs via caspase 3/7 activation, and both p53-dependent and -independent mechanisms have been observed [18, 19, 25, 31–44]. Thus, it may be that progressive muscle loss results from stochastic activation of *DUX4* over time. Yet, the mechanism of how DUX4 induces apoptosis remains unclear. Many DUX4-dependent cytopathologies have been documented, including oxidative stress [45], DNA damage [46], impaired ubiquitin-dependent proteolysis [47], defective RNA quality control [48, 49], altered splicing [18], nuclear protein aggregation [36, 47], nuclear double-stranded RNA aggregates [44], altered mitochondrial metabolism [50], and aberrant nuclear import/export [51], which may all contribute to cell death. Additionally, much evidence indicates that DUX4 expression causes mis-regulation of signal transduction pathways including hyaluronic acid [36], hypoxia [41], β-catenin [52, 53], innate immunity [44], RET [54], and MAP kinase [55] pathways. As a result, most FSHD therapeutic development have focused on DUX4-targeting therapies, either using CRISPR-based approaches [56–59], or antisense oligonucleotide/nucleic acid-based approaches to target the transcript [60–69]. Alternatively, small molecule-based approaches have been proposed that inhibit activation of the *DUX4* gene [70] with losmapimod currently undergoing clinical trial (ClinicalTrials.gov Identifier: NCT04003974).

An alternate approach is to target DUX4-induced cytopathologies that trigger apoptosis. We have previously demonstrated that inhibiting hyaluronic acid signaling [36] or inhibition of the mTor/PI3K/AKT pathway [41] can ameliorate DUX4-induced toxicity in myoblasts. Additionally, it has recently been demonstrated that inhibition of MAP kinase pathways can have a similar effect [55]. We set out to leverage these observations to identify compounds that can inhibit DUX4-induced toxicity and may be developed into FSHD therapeutics. To this end, we performed two rounds of screening and characterization on compounds that were selected either due to their structural similarity to the hyaluronic acid synthesis inhibitor 4MU or its metabolite 4MUG, or based on previous reports of activity in relevant signal transduction pathways. We identified a set of five flavones that can inhibit DUX4-induced toxicity at low-micromolar concentrations, thereby making them candidates for further therapeutic development. Additionally, we show that these compounds function through an mTor/AKT-independent mechanism that results in activation of cellular autophagy, thereby demonstrating that targeting both mTor-dependent and mTor-independent biochemical pathways are viable approaches for FSHD therapeutic development, and specifically identifying autophagy as a novel target for therapies.

## Results

### First-generation small molecules inhibit DUX4-induced apoptosis

**Fig. 1:**
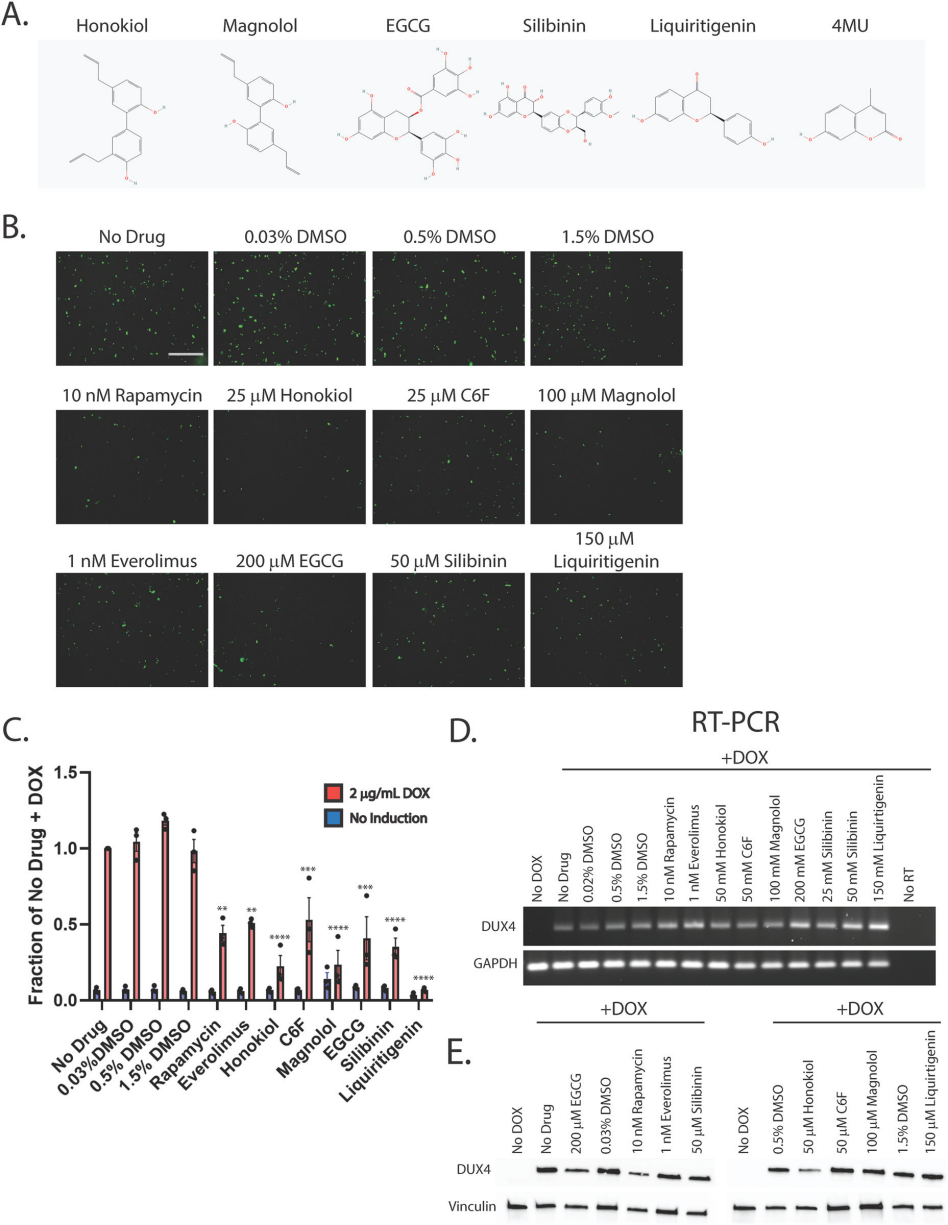
First-generation compounds provide resistance to DUX4-induced toxicity. **A** Pubchem [81] structures of compounds under study. 4MU is also shown for comparison to the structures of EGCG, silibinin, and liquiritigenin. **B** MB135-DUX4i myoblasts were pre-treated with the indicated compounds for 3 h, followed by addition of 2 μg/mL doxycycline (DOX) to the media to induce DUX4 expression. After 24 h, caspase 3/7 activation was visualized using the CellEvent Caspase-3/7 Green reagent. Scale bar = 400 μm. **C** MB135-DUX4i myoblasts were treated as in B. and cell death was quantified using a Caspase-Glo 3/7 Assay. Statistical significance for doxycycline-induced drug-treated samples vs relevant doxycycline-induced vehicle-treated controls is shown and was calculated using 1-way ANOVA with Tukey’s test. *P < 0.05, **P < 0.01, ***P < 0.001, ****P < 0.0001. Error bars are SEM. **D** Myoblasts were treated as in (**B**) and were then analyzed for DUX4 induction using limited-cycle RT-PCR. **E** Myoblasts were treated as in (**B**) and DUX4 protein expression was measured by western blotting with the DUX4 E55 antibody. Vinculin was also included as a loading control. Uncropped blot images are presented in the Supplementary Information.

**Fig. 2:**
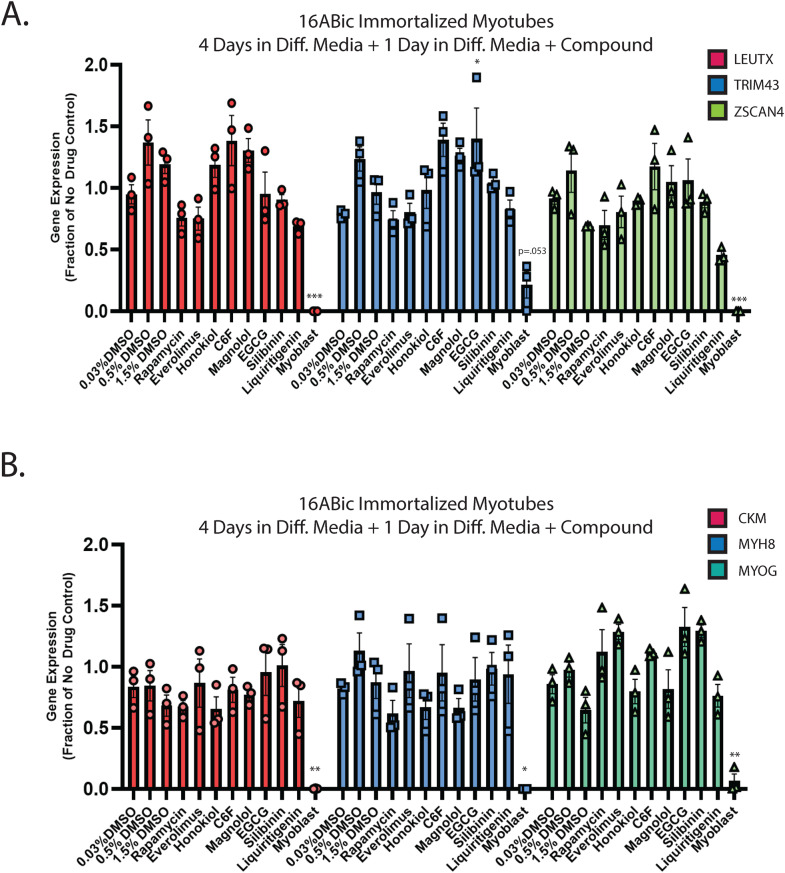
First-generation compounds have minimal impact on DUX4 activity or expression of myogenic marker genes in patient-derived myotubes. **A** Immortalized 16ABic myoblasts were seeded to high density on gelatin coated 6-well plates. The following day, growth media was replaced with differentiation media, and differentiation was allowed to proceed for 4 days. Media was then replaced with fresh diff. media containing the indicated compound and myotubes were incubated for an additional 24 h, followed by quantification of expression of three DUX4-target genes by qRT-PCR. **B** As in (**A**), but qRT-PCRs were performed with primers specific to markers of myogenesis. Error bars are SEM. Statistical significance for samples vs relevant vehicle-treated controls is shown and was calculated using 1-way ANOVA with Tukey’s test. *P < 0.05, **P < 0.01, ***P < 0.001.

In our previous work, we demonstrated that the hyaluronic acid synthesis inhibitor 4MU can provide resistance to DUX4-induced toxicity [36]. Additionally, we and others have implicated hypoxia signaling as central to toxicity [41, 52]. Interestingly, these signaling pathways converge on the mTor/AKT/PI3K signal transduction axis [71, 72], and inhibition of this pathway can provide resistance to toxicity [41]. We sought to leverage these observations to identify small molecules that can provide resistance to DUX4-induced apoptosis. Unfortunately, 4MU itself requires millimolar doses for maximal effectiveness, and so is not suitable for use as a therapeutic. Thus, we considered other molecules that may provide a similar result at lower concentrations. Based on previous reports of their activity in a relevant pathway, we identified a panel of six first-generation compounds that had the potential to meet this criteria- honokiol (CAS # 35354-74-6), its synthetic analogue claisened hexafluoro (C6F) [73, 74], magnolol (CAS # 528-43-8), epigallocatechin gallate (EGCG, CAS # 989-51-5), silibinin (CAS # 22888-70-6), and liquiritigenin (CAS # 69097-97-8) [75–79]. Notably, EGCG, silibinin, and liquiritigenin were of particular interest because their chemical structures are built around the same fused ring structure as 4MU, and they each maintain the chemically active hydroxyl group [80, 81] (Fig. 1A). To evaluate these compounds, we used the MB135-DUX4i myoblast model [38]. Myoblasts were seeded on 96 well plates, and the following day they were pre-treated by adding the indicated compound to the media for 3 h, followed by the addition of 2 μg/mL doxycycline (DOX) to the media for 24 h to induce DUX4 expression (for a total of 27 h of exposure to the compounds). As a positive control, we also included the mTor inhibitor rapamycin, which can inhibit DUX4-induced toxicity [41] and its next-generation analogue everolimus. Cell death was then visualized using the CellEvent Caspase 3/7 Green assay (Invitrogen, Waltham, MA USA). We tested a range of concentrations and found that each was able to provide resistance to toxicity when administered at proper concentrations (Figs. 1B, S1). To confirm and quantitate these results, we conducted similar experiments using the Caspase-Glo 3/7 assay system (Promega, Madison, WI USA). Each compound provided at least a twofold reduction in caspase 3/7 activity relative to vehicle controls, with liquiritigenin showing the strongest effect (Fig. 1C). To validate these results we performed limited-cycle RT-PCR as described previously [41] and confirmed that these compounds did not interfere with the induction of the codon-altered DUX4 transgene (Fig. 1D). Similarly, we performed western blotting analysis to determine the effects on the levels of DUX4 protein. As observed previously [41], rapamycin caused a drop in the abundance of DUX4 (Fig. 1E). Surprisingly however, only honokiol showed a similar decline in DUX4 protein abundance, but the remaining compounds had no effect. This confirms that the mechanism of observed resistance to DUX4-induced toxicity occurs downstream of DUX4 expression, but also unexpectedly suggests that these compounds function via a different mechanism than rapamycin. Finally, to confirm that these compounds function downstream of DUX4 expression and that they do not have a deleterious effect on mature myotubes, immortalized patient-derived 16ABic myoblasts [82] were induced to form myotubes for 4 days using established methods [83], and were then treated with compounds for 24 additional hours. The expression of three DUX4-target genes were then analyzed using qRT-PCR, and no significant change was observed (Fig. 2A). We also analyzed three myogenesis markers and found no statistically significant effect on their expression (Fig. 2B). Similar results were observed using a second patient-derived cell line (Fig. S2).

### Second-generation small molecules are more potent inhibitors of DUX4-induced apoptosis

**Fig. 3:**
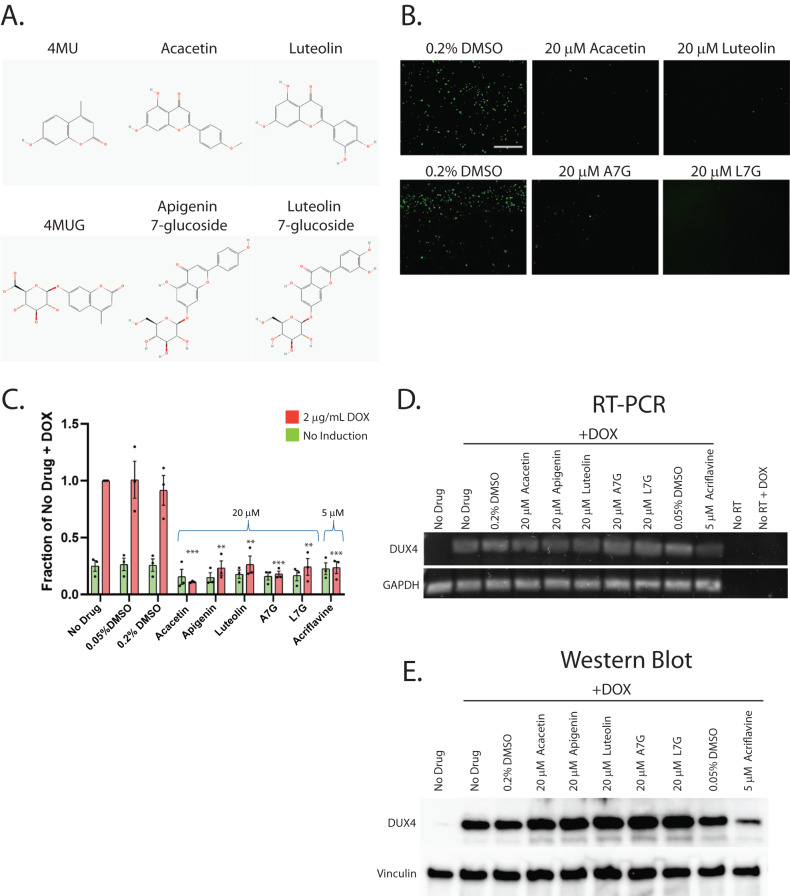
Second-generation compounds provide resistance to DUX4-induced toxicity. **A** Pubchem [81] structures of selected compounds under study as well as 4MU and 4MUG for comparison. **B** MB135-DUX4i myoblasts were pre-treated with the indicated compounds for 3 h, followed by addition of 2 μg/mL doxycycline to the media to induce DUX4 expression. After 24 h, caspase 3/7 activation was visualized using the CellEvent Caspase-3/7 Green reagent. The screen was performed twice, each time in triplicate, for a total of six independent replicates. The full screen is presented in Figs. S3 and S4. Scale bar = 400 μm. **C** MB135-DUX4i myoblasts were treated as in (**B**) and cell death was quantified using a Caspase-Glo 3/7 Assay. Statistical significance for doxycycline-induced drug-treated samples vs relevant doxycycline-induced vehicle-treated controls is shown and was calculated using 1-way ANOVA with Tukey’s test. *P < 0.05, **P < 0.01, ***P < 0.001. Error bars are SEM. **D** Myoblasts were treated as in (**B**) and were then analyzed for DUX4 induction using limited-cycle RT-PCR. **E** Myoblasts were treated as in (**B**) and DUX4 protein expression was measured by western blotting with the DUX4 E55 antibody. Uncropped images of blots are presented in the Supplementary Information. A7G, Apigenin 7-glucoside. L7G, Luteolin 7-glucoside.

**Fig. 4:**
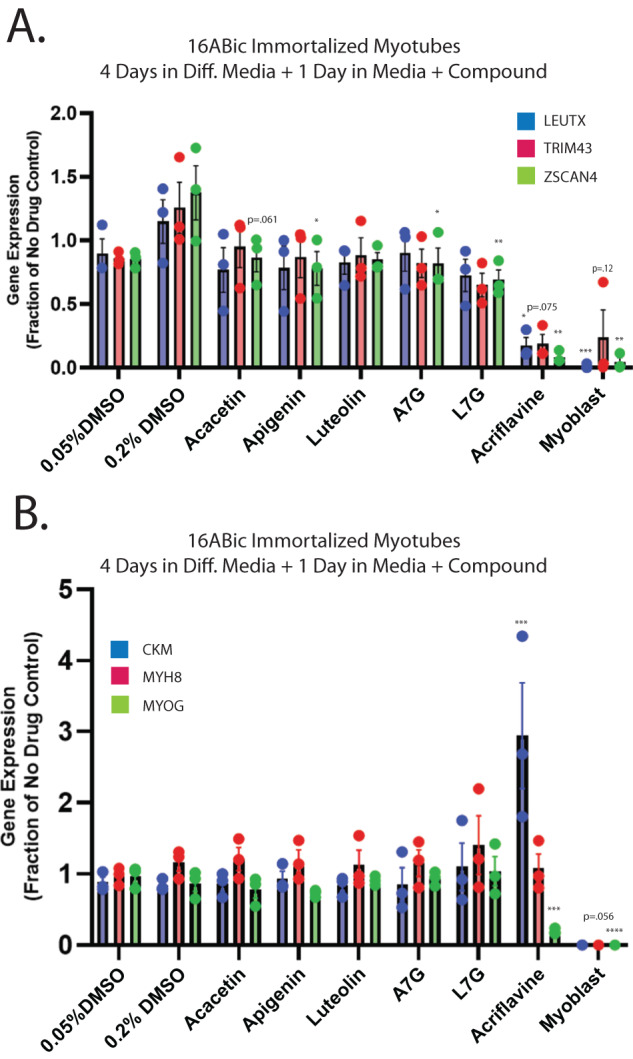
Second-generation compounds have minimal impact on DUX4 activity or expression of myogenic marker genes in patient-derived myotubes. Patient-derived myoblasts were analyzed as in Fig. 2, but treated with second generation compounds, followed by qRT-PCR analysis using (**A**). DUX4-target gene or (**B**). myogenic marker gene primer sets. Error bars are SEM. Statistical significance for samples vs relevant vehicle-treated controls is shown and was calculated using 1-way ANOVA with Tukey’s test. *P < 0.05, **P < 0.01, ***P < 0.001, ****P < 0.0001. A7G Apigenin 7-glucoside, L7G Luteolin 7-glucoside.

While our first-generation compounds were effective at inhibiting DUX4-induced toxicity, the most potent, liquiritigenin, required a 150 μM dose for optimal effectiveness. While this is a significant improvement relative to concentrations required for 4MU, it is still too high for therapeutic use. To overcome this limitation, we performed a second screening of a larger library of compounds (Table 1). Many of these were additional flavone compounds that bear structural similarity to 4MU or to its bioactive metabolite 4MUG [84] (Fig. 3A). For the initial characterization, we used the protocol described above using 50, 5, or 1 μM concentrations and the CellEvent assay (Fig. S3). The best performing compounds were then selected for a secondary screening /optimization using concentrations of 30, 20, or 10 μM (Fig. S4). This screen identified 5 compounds- acacetin (CAS # 480-44-4), apigenin (CAS # 520-36-5), luteolin (CAS # 491-70-3), apigenin 7-glucoside (A7G, CAS # 578-74-5), and luteolin 7-glucoside (L7G, CAS # 5373-11-5), which provided resistance to DUX4-induced toxicity at optimal concentrations of 20 μM (Fig. 3B). We also identified acriflavine (CAS # 8048-52-0), which based on phase-contrast images provided resistance at 5 μM (Fig. S3). We again quantitated these results using the Caspase-Glo assay and found that each compound provided at least four-fold reduction in DUX4-induced toxicity (Fig. 3C). As before, we validated these results using limited-cycle RT-PCR. Acriflavine showed a notable decline in DUX4 transcript levels, suggesting that its effects were an artifact of inhibited transgene activation. Other compounds did not have an effect an *DUX4* expression (Fig. 3D). Similarly, western blotting showed that the second-generation compounds did not cause a post-transcriptional decline in DUX4 protein levels (Fig. 3E). This again suggests that these compounds exert their effect through a different mechanism than rapamycin. We also examined the effects of these compounds in patient-derived myotubes, and we again found that there was no change in DUX4-target gene expression levels, except for acriflavine, which inhibited expression of all three DUX4 target genes significantly (Fig. 4A), however this appears to be an artifact of acriflavine disrupting myogenesis, as it caused significant overexpression of CKM, while also inhibiting MYOG expression. Similar results were observed using a second patient-derived cell line (Fig. S5). These results are again consistent with the five flavone compounds functioning downstream of DUX4 to inhibit toxicity without having negative effects on myogenesis.

**Table 1:** Second-Generation Compound Screen.

Compound	CAS Number	Group
Acacetin	480-44-4	4MU-like
Apigenin	520-36-5	4MU-like
Chrysin	480-40-0	4MU-like
Farrerol	95403-16-0	4MU-like
Fisetin	528-48-3	4MU-like
Isorhamnetin	480-19-3	4MU-like
Kaempferol	520-18-3	4MU-like
Luteolin	491-70-3	4MU-like
Myricetin	529-44-2	4MU-like
Quercetin	117-39-5	4MU-like
Scutellarein	529-53-3	4MU-like
4MUG	881005-91-0	4MUG-like
Apigenin 7-glucoside (A7G)	578-74-5	4MUG-like
Liquiritin	551-15-5	4MUG-like
Luteolin 7-glucoside (L7G)	5373-11-5	4MUG-like
Myricitrin	17912-87-7	4MUG-like
Vitexin	3681-93-4	4MUG-like
Scutellarin	27740-01-8	4MUG-like
Acriflavine	8048-52-0	Misc.
Imatinib	152459-95-5	Misc.
Londamine	50264-69-2	Misc.
Oxythiamine	136-16-3	Misc.
Mitoquinone (MitoQ)	845959-50-4	Misc.
PT2385	1672665-49-4	Misc.
Shikonin	517-89-5	Misc.
TAT-cyclo-CLLFVY	1446322-66-2	Misc.

A list of compounds tested in the second-generation compound screen.

### Flavones and rapamycin inhibit DUX4-induced toxicity through distinct mechanisms

**Fig. 5:**
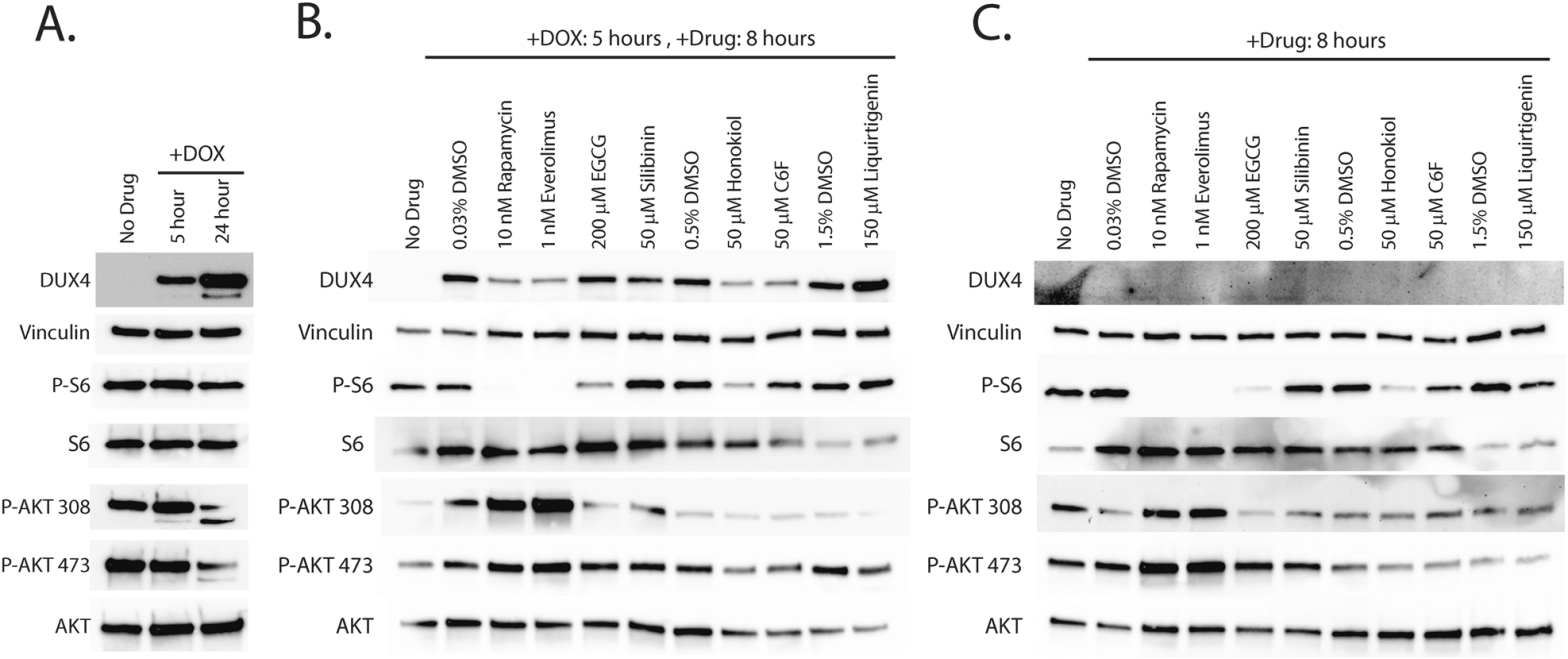
Compounds prevent DUX4-induced toxicity through distinct mechanisms. **A** MB135-DUX4i myoblasts were either left un-induced or induced with doxycycline for 5 or 24 h. At the indicated times myoblasts were lysed and analyzed by western blotting with antibodies specific to markers of the mTor/AKT signaling axis. **B** MB135-DUX4i myoblasts were pre-treated with the indicated concentrations of compounds for 3 h, followed by induction of DUX4 expression for 5 h and western blotting analysis as in (**A**). **C** MB135-DUX4i myoblasts were left un-induced and exposed to the indicated concentrations of compounds for 8 h, followed by western blotting analysis as in (**A**). Uncropped images of all blots are presented in the Supplementary Information. P-S6 phosphorylated S6 protein, P-AKT 308 AKT phosphorylated at Thr308, P-AKT 473 AKT phosphorylated at Ser473.

We next investigated the mechanism of action of the flavones. First, we used phospho-specific antibodies to determine how activation of DUX4 affects signaling in the mTor/AKT pathway. We analyzed either uninduced MB135-DUX4i myoblasts, or myoblasts induced with 2 μg/mL doxycycline for 5 or 24 h with antibodies specific to AKT phosphorylated on Thr308 or Ser473, or phosphorylated ribosomal S6 protein, a marker of mTor activity. After 5 h of induction, we observed no change in phosphorylation of S6 or AKT at Ser473, and only a small but reproducible increase in AKT phosphorylation at Thr308 (Fig. 5A). However, we observed a notable decline in phosphorylation of AKT after 24 h of DUX4 expression. In contrast, there was no change in the levels of overall AKT or S6 protein. Thus, it may be that prolonged DUX4 expression inhibits signaling along this axis, or that signaling along the mTor/AKT axis is lost at this late time point, as these populations are actively undergoing apoptosis. To determine the effects of our compounds on AKT/mTor activation, we pre-treated myoblasts with compounds for 3 h and then induced DUX4 expression for 5 h. As expected, rapamycin and everolimus ablated S6 phosphorylation and triggered hyperphosphorylation of AKT, particularly on Thr308 (Fig. 5B). Surprisingly, of the compounds under study, only honokiol showed a reproducible, but partial inhibition of S6 phosphorylation, and none showed hyperphosphorylation of AKT. Thus, with the possible exception of honokiol, it appears that the first-generation compounds inhibit toxicity through an mTor-independent mechanism. Interestingly, the effects are not DUX4-specific, as performing the same experiments without inducing DUX4 yielded nearly identical results (Fig. 5C), suggesting that they function not by inhibiting a DUX4-activated signal transduction pathway, but rather by triggering a DUX4-independent response that protects against toxicity. To confirm that these observations also hold true for the second-generation compounds, we also pretreated MB135-DUX4i myoblasts with luteolin or L7G for 3 h and either left them uninduced for five more hours or induced them with doxycycline for five or 24 h and analyzed by western blotting (Fig. S6). As before, the second-generation compounds showed no inhibition of S6 phosphorylation.

### Second-generation compounds prevent loss of ULK1 and induce a marker of autophagy

**Fig. 6:**
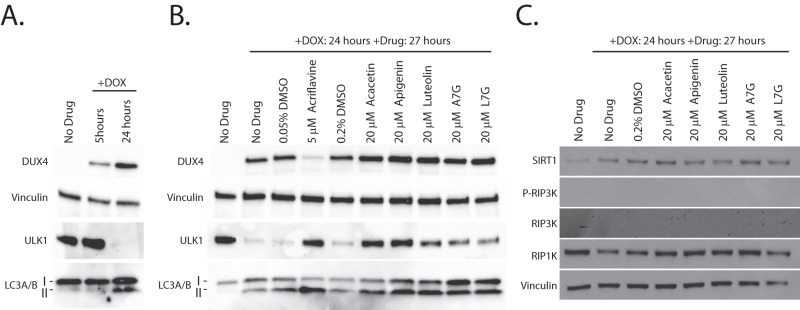
Flavones protect ULK1 and cause increased levels of the autophagy marker LC3-II. **A** MB135-DUX4i myoblasts were either left un-induced or induced with doxycycline for 5 or 24 h. At the indicated times myoblasts were lysed and analyzed by western blotting with antibodies specific to markers of autophagy. **B** MB135-DUX4i myoblasts were pre-treated with the indicated concentrations of compounds for 3 h, followed by induction of DUX4 expression for 24 h and western blotting analysis as in (**A**). Uncropped images of all blots are presented in the Supplementary Information. **C** MB135-DUX4i myoblasts were treated as in (**B**) and were then analyzed by western blotting with antibodies specific to RIPK1, RIPK3, phospho-RIPK3, and SIRT1.

To investigate the mechanism of action of the second-generation flavone compounds, we interrogated other pathways that may protect against DUX4-induced toxicity. We considered that the antioxidant activity of the flavones may be responsible for their protective effect, as FSHD pathology is associated with oxidative stress [46, 50, 85] and antioxidants have been shown to inhibit DUX4-induced toxicity in DUX4-expressing C2C12 cells [32, 86]. However, similar to our previous results [36], we found the antioxidant coenzyme Q10 did not prevent DUX4-induced toxicity and could not prevent the mis-localization of the DUX4-interacting protein C1QBP (a marker of pathology) (Fig. S7). While the closely related MitoQ did inhibit toxicity, this was due to decreased of DUX4 expression (Figs. S3, S7). Alternatively, we considered that flavones may function by modulating autophagic activity, as luteolin has been previously reported as both a positive and negative regulator of autophagy [87], suggesting that this pathway may be relevant. Furthermore, rapamycin is a known autophagy activator [88], and autophagy regulation integrates multiple signaling pathways [89]. Therefore, both mTor-dependent and mTor-independent mechanisms that regulate autophagy may affect DUX4-induced toxicity. We investigated this possibility by analyzing ULK1 expression, a key autophagic regulator that integrates multiple signaling pathways [89]. After 5 h of induction there was no notable change in the levels of ULK1 protein (Fig. 6A). However, after 24 h much of ULK1 protein was lost. Surprisingly, the decline in ULK1 was accompanied by an increase in LC3-II, a marker of active autophagy. To investigate the effects of the second-generation compounds on autophagy, we pre-treated myoblasts for 3 h and then induced DUX4 for 24 h. We found that the flavone compounds protected ULK1 from DUX4-induced loss. This effect appears to be specific to ULK1 and not a general property of the autophagic machinery, as DUX4 induction did not cause loss of the autophagy-associated proteins ATG3, ATG5, ATG7, or ATG16L1, and the flavones had no effect on these proteins (Fig. S8). Interestingly, we observed that each of the flavones increased the abundance of the LC3-II autophagy marker well above the level induced by DUX4 alone (Fig. 6B), suggesting that cellular autophagy protects against DUX4-induced toxicity and that the flavone compounds function by enhancing this protective mechanism. Finally, we asked whether the flavones function in mTor-independent pathways that are known to modulate autophagic activity. One candidate pathway is necroptosis, which functions via RIPK1 or RIPK3 activation, is regulated by ULK1 [90], and has recently been shown to contribute to DUX4-induced toxicity [43]. A second possible pathway is via SIRT1, which can directly activate autophagy via an mTor- and ULK1-independent pathway [91], and is known to be activated by some flavones [92]. To address these possibilities, we treated MB135-DUX4i cells with second generation compounds and induced DUX4 expression as above and analyzed lysates by immunoblotting. We did not detect RIP3K in MB135-DUX4i myoblasts, consistent with its minimal expression in human skeletal muscle (https://www.gtexportal.org/home/gene/RIPK3), and we detected no reproducible changes in RIPK1. However, we did observe that DUX4 induction caused a reproducible increase in the levels of SIRT1 (Fig. 6C). This raised the possibility that the DUX4-induced increase in autophagy may be mediated by increased SIRT1 levels, and that the flavones may produce resistance to toxicity by modulating SIRT1 activity. To test this hypothesis, we treated the model myoblasts with either the strong SIRT1 activator SRT1720 (Fig. S9), or the SIRT1 inhibitor Selisistat (Fig. S10). We found that neither SIRT1 activation nor inhibition had any effect on DUX4-induced toxicity, and that at higher concentrations both caused DUX4-independent toxicity. Taken together, these observations reenforce a model where flavone compounds protect from DUX4-induced toxicity by promoting autophagy via a ULK1-dependent mechanism, identify flavones in particular as potential drugs for further development, and identify the autophagy pathway in general as a target for future FSHD therapeutics.

## Discussion

While significant advancement in the characterization of DUX4-induced cytopathologies has been made, the precise mechanism that leads to DUX4-induced apoptosis has remained elusive. Increasing evidence indicates that DUX4 causes widespread mis-regulation of signaling pathways [36, 41, 44, 52–55], making these cascades important potential targets for FSHD therapeutics. The mTor/AKT signaling axis is of particular interest as it mediates signaling through both the hyaluronic acid and HIF1α pathways. Additionally, mTor regulates energy homeostasis, and we and others have seen mitochondrial anomalies in response to DUX4 expression and in FSHD muscle [36, 50, 85, 93–95], and a recent study has demonstrated metabolic disruption in FSHD [50]. Despite this, most therapeutic development have focused on targeting *DUX4* directly. Thus, in this study we endeavored to identify inhibitors of DUX4-induced toxicity that function downstream of DUX4. We conducted two screens of compounds predicted to function in the mTor/AKT/HIF1α pathway, and we identified three first-generation and five second-generation flavone/flavonoid compounds that inhibit DUX4-induced toxicity (Figs. 1, 3). Importantly, the second-generation compounds function at pharmacologically relevant concentrations, and none have negative effects on the expression of myogenic markers in FSHD patient-derived myotubes (Figs. 4, S5). These compounds therefore have the potential to be investigated further as treatments for FSHD.

Unexpectedly, we have also observed that the flavones do not function through the mTor/AKT axis. While rapamycin and everolimus ablated the phosphorylation of the S6 ribosomal protein and caused hyperphosphorylation of AKT, the flavones did not show either of these effects. Rather, we observed that the flavones protected ULK1, a regulator of autophagy [89] from DUX4-induced loss (presumably by triggering its degradation) (Fig. 6A, B). Importantly, this correlated with an increase in the autophagy marker LC3-II (Fig. 6B), which implies that the flavone compounds protect against the toxic effects of DUX4 expression by inducing autophagy. It remains unknown how these compounds activate autophagy, however a plausible explanation is that they function via activation of AMPK. Luteolin has been implicated in activating AMPK in muscle previously [96], and AMPK and mTor have opposing effects on autophagy [89]. Therefore, rapamycin and flavones may ultimately share a separate, but converging mechanism of action, but this hypothesis requires a dedicated study to confirm.

The unexpected mTor-independent mechanism of action of the flavones potentially makes them a particularly interesting alternative therapeutic to mTor inhibitors. While compounds such as rapamycin can cause reduced DUX4 protein levels, they are also potent protein synthesis inhibitors that could prevent generation of new muscle protein and contribute to muscle pathology [97, 98]. Furthermore, rapamycin is also an immunosuppressant, which would create an additional burden on patients. Additionally, flavones may provide benefits to patients independent of their effect on DUX4-induced toxicity, as a recent publication demonstrated that closely-related isoflavone compounds can correct the “hypotrophic” phenotype of FSHD myotubes [93]. This family of compounds therefore has great therapeutic potential for FSHD.

The protective effect observed here may have implications for FSHD research beyond their therapeutic potential. There is a significant body of literature indicating that FSHD genetic markers do not strictly correlate with the disease phenotype, and DUX4 expression has been detected in the muscle of unaffected individuals (reviewed in [2]). Thus, it has long been hypothesized that either additional unknown genetic factors can modify the disease phenotype, or that environmental factors influence progression (or a combination of both). Flavones may be one such environmental factor, as they can be introduced via the diet [99]. Different flavones can have very different effects on DUX4-induced toxicity (Fig. S3), and so it is possible that diets rich in a particular flavone may have notable effects while others are negligible, and that this may contribute to disparate disease progression. Detailed studies of diets rich in the most effective flavones or flavone supplementation will be necessary to determine if these are viable approaches to control disease progression.

## Methods

### Cell culture

MB135-DUX4i myoblasts were grown essentially as described previously [36] in MB135 media (20% FBS, 1% antibiotic antimycotic, 10 ng/mL FGF [Gibco PHG0026], 10 μM dexamethasone [Sigma, St. Louis, MO, D4902] in Ham’s F10 media). Immortalized 16ABic and 01ABic [82] myoblasts were grown on gelatin-coated (Sigma G9391) plates in HMP media (20% FBS, 1% antibiotic/antimycotic, 1.2 mM CaCl_2_, 0.5% chick embryo extract [MP Biomedicals, Santa Ana, CA, 092850145]). Myotubes were generated by plating 4-6X10^5^ myoblasts per well on gelatin-coated 6-well plates. The following day cells were washed with PBS, and media was replaced with 15% KOSR differentiation media, essentially as described [83] (glutamax substituted for L-glutamine), and allowed to differentiate for 4 days. Media was then replaced and supplemented with the relevant compound for 24 h. All experiments were performed on three independently grown cell cultures unless otherwise noted.

### Caspase assays

15,000 myoblasts/well were plated on 96-well plates. The next day media was replaced with media containing the indicated compounds for the stated times. 24 h after doxycycline induction 5 μL of CellEvent Caspase-3/7 Green reagent (Invitrogen R37111) was added per well, plates were incubated for 30 min at 37 °C, and were then imaged with an Echo Revolve microscope or a Nikon Eclipse TS100 inverted microscope (Figs. S9, S10). Where applicable, Hoechst staining was performed at 1 μg/ml. For quantitative assays, the Caspase-Glo® 3/7 Assay (Promega G8090 or G8091) was performed in duplicate (two wells were measured for each of 3 replicates, for a total of 6 wells measured per condition) according to the manufacturer’s instructions, and luminescence was measured in a BioTek Synergy LX multi-mode plate reader.

### Gene expression analysis

Cells were grown on 6-well plates as described above and were lysed in Buffer RLT with 2-mercaptoethanol, scraped, moved into a 1.5 mL tube, homogenized by pipetting with a P200 tip >60 times, followed by freezing at −80 °C. Total RNA was extracted with an RNEasy Mini kit (Qiagen, Venlo, Netherlands, 74106) with on-column DNase I (Qiagen, 79254) digestion. cDNA was made from up to 1 μg of total RNA with a SuperScript III first-strand synthesis kit (Invitrogen, 18080051) with double priming, and the RNase H step was performed. Limited-cycle PCR was performed as described [41]. qPCR was performed as described [36] using published primers [23, 36, 100] (Table S1). All experiments were performed in triplicate.

### Protein expression analysis

Myoblasts were grown on 6-well plates as described, washed in PBS, lysed, and scraped in 150 μL RIPA (Pierce/Thermo Scientific Waltham, MA USA, 89900) supplemented with PhosSTOP (Roche Penzberg, Germany, 04 906 837 001) and 1% protease inhibitor cocktail (Sigma P8340). Cells were lysed at 4 ^o^C for 20 min, centrifuged at 12,000*g* for 15 min, and the pellet was discarded. Protein concentrations were measured by BIO-RAD DC assay, and 5–10 μg of protein was run. Primary antibodies and dilutions: DUX4 E55 1:1000 (AbCam, Cambridge, UK, ab124699), Vinculin 1:10,000 (Sigma V9131), phosphorylated ribosomal protein S6 1:1000 (Cell Signaling Danvers, MA USA, 4858S), ribosomal protein S6 1:1000 (Cell Signaling 2317S), phosphorylated AKT 308 1:1000 (Cell Signaling 13038P), phosphorylated AKT 473 1:1000 (Cell Signaling 4060S), Pan-AKT 1:1000 (Cell Signaling 4691P), ULK1 1:1000 (Cell Signaling 8054T/S), and LC3A/B 1:1000 (Cell Signaling 12741T), SIRT1 1:1000 (Cell Signaling 9475), RIP3 1:1000 (Cell Signaling 10188), Phospho-RIP3 (Ser227) 1:1000 (Cell Signaling 93654), RIP 1:000 (Cell Signaling 3493). Secondary antibodies and dilutions: anti-rabbit and anti-mouse IgG-HRP 1:2000 (Cell Signaling 7074P2 and 7076S). ECL reagents: Clarity Western ECL Substrate (BIO-RAD, Hercules, CA 1705060) and Clarity MAX Western ECL Substrate (BIO-RAD1705062). Blots were visualized on a BIO-RAD ChemiDoc or ChemidocMP with Universal Hood III. Uncropped versions of all western blots are presented in the Supplementary Materials.

### Immunostaining

Myoblasts were fixed and stained as previously described [36] using 1:400 anti-DUX4 antibody P2B1 (Invitrogen MA5-27584) and 1:300 anti-C1QBP (Bethyl Laboratories, Montgomery Texas, USA A302-862A).

### Supplementary information

Supp. Figures

Supp. Figure and Table Legends

Table S1: Primers

Checklist

## Data Availability

All data necessary to support the conclusions of the paper are displayed in the figures or Supplementary Materials.
