# Supplementary material for: Flavones provide resistance to DUX4-induced toxicity via an mTor-independent mechanism
Source: Cell Death Dis. 2023 Nov 16;14(11):749. doi: 10.1038/s41419-023-06257-2 (PMC10654915; doi:10.1038/s41419-023-06257-2)
Supplement: Supplementary file 2 — Supp. Figure and Table Legends [file 41419_2023_6257_MOESM2_ESM.docx]

**Supplementary Figure Legends**

**Figure S1: Optimization of First-generation compounds.** MB135-DUX4i myoblasts were pre-treated with the indicated compounds for 3 hours, followed by addition of 2 μg/mL doxycycline to the media to induce DUX4 expression. After 24 hours, caspase 3/7 activation was visualized using the CellEvent Caspase-3/7 Green reagent. The optimization was performed twice, each time performed in triplicate, for a total of six independent replicates. Scale bar = 400 μm.

**Figure S2: First-generation compounds have minimal impact on DUX4 activity or expression of myogenic marker genes in patient-derived myotubes.** Analysis of the effects of first-generation compounds on **A.** DUX4-target gene expression or **B.** myogenic marker gene expression was performed as in Figure 2 using immortalized 01ABic myogenic cells. Error bars are SEM. Statistical significance for samples vs relevant vehicle-treated controls is shown and was calculated using 1-way ANOVA with Tukey’s test. *P<0.05, **P<0.01, ****P<0.0001.

**Figure S3: First screen/titration of second-generation compounds.** MB135-DUX4i myoblasts were treated as in Figure 1B. Three concentrations of each compound being screened were used as well as equal volume DMSO vehicle controls. Caspase 3/7 activation was visualized using the CellEvent Caspase-3/7 Green reagent. Luteolin is partially fluorescent in the relevant channel, creating significant background at high concentrations. Acriflavine is highly fluorescent in the relevant channel, and so phase-contrast images are presented instead. The screen was performed twice, each time in triplicate, for a total of six independent replicates. **A.-C.** The screen was broken into three sub-screens. Each panel represents one replicate of a particular sub-screen. Scale bar = 400 μm. A7G, Apigenin 7-glucoside. L7G, Luteolin 7-glucoside. 4MUG, 4-methylumbelliferyl glucuronide. MitoQ, mitoquinone.

**Figure S4: Optimization of second-generation compounds.** The most effective compounds from Figure S3 were selected for further optimization. Experiments were performed exactly as in Figure S3, but with a more narrow range of concentrations. **A.-C.** As Figure S3, screens were divided into 3 sub-screens performed separately. The screen was performed twice, each time in triplicate, for a total of six independent replicates. Each panel is one representative replicate. **D.** Titrations of acriflavine were visualized by phase-contrast microscopy. Scale bar = 400 μm. A7G, Apigenin 7-glucoside. L7G, Luteolin 7-glucoside.

**Figure S5:** **Second-generation compounds have minimal impact on DUX4 activity or expression of myogenic marker genes in patient-derived myotubes.** Analysis of the effects of second-generation compounds on **A.** DUX4-target gene expression or **B.** myogenic marker gene expression was performed as in Figure 4 using immortalized 01ABic myogenic cells. Error bars are SEM. Statistical significance for samples vs relevant vehicle-treated controls is shown and was calculated using 1-way ANOVA with Tukey’s test. *P<0.05, **P<0.01. A7G, Apigenin 7-glucoside. L7G, Luteolin 7-glucoside.

**Figure S6:** **Second-generation compounds do not inhibit mTor activity.** MB135-DUX4i myoblasts were pre-treated with the indicated compounds for 3 hours followed by an additional 24 hours either with or without doxycycline induction of DUX4 expression, followed by western blotting with the indicated antibodies. This experiment was performed twice. Uncropped images of all blots are also presented in the supplemental information. A7G, Apigenin 7-glucoside. L7G, Luteolin 7-glucoside. P-S6, phosphorylated S6 protein.

**Figure S7: An antioxidant does not prevent DUX4-induced pathology.** MB135-DUX4i myoblasts were induced to express DUX4 and were simultaneously treated with the indicated concentration of coenzyme Q10 (CoQ10), mitoquinone (MitoQ), or an equal volume of DMSO vehicle. 24 hours after induction, cells were fixed and stained with antibodies specific to DUX4 and C1QBP.

**Figure S8:** **ATG proteins are not affected by DUX4 expression or flavones.** MB135-DUX4i myoblasts were pre-treated with the indicated concentrations of compounds for 3 hours, followed by induction of DUX4 expression for 24 hours and western blotting analysis with antibodies specific to ATG3, ATG7, or the loading control vinculin. Uncropped images of all blots are also presented in the supplemental information. A7G, Apigenin 7-glucoside. L7G, Luteolin 7-glucoside.

**Figure S9: SIRT1 activation does not prevent DUX4-inducted pathology.** MB135-DUX4i cells were treated with the indicated concentration of SRT1720 for 3 hours followed by induction of DUX4 expression for 24 hours. After 24 hours, caspase 3/7 activation was visualized using the CellEvent Caspase-3/7 Green reagent (Caspase 3/7) and nuclei were stained with Hoechst dye (Hoechst).

**Figure S10: SIRT1 inhibition does not prevent DUX4-inducted pathology.** MB135-DUX4i cells were treated with the indicated concentration of Selisistat for 3 hours followed by induction of DUX4 expression for 24 hours. After 24 hours, caspase 3/7 activation was visualized using the CellEvent Caspase-3/7 Green reagent (Caspase 3/7) and nuclei were stained with Hoechst dye (Hoechst).

**Supplementary Tables**

**Table S1: Primers**: Sequences of primers used in this study.
