# Supplementary material for: Flavones provide resistance to DUX4-induced toxicity via an mTor-independent mechanism
Source: Cell Death Dis. 2023 Nov 16;14(11):749. doi: 10.1038/s41419-023-06257-2 (PMC10654915; doi:10.1038/s41419-023-06257-2)
Supplement: Supplementary file 3 — Table S1: Primers [file 41419_2023_6257_MOESM3_ESM.docx]

**Table S1: Primers**

| Primer pair | Sequences | Reference |
| --- | --- | --- |
| LEUTX | For: CAAGAAGGGCCAAGGCGTTA  Rev: GCCTCTTCCATTTGGCACGC | 36 |
| TRIM43 | For: ACCCATCACTGGACTGGTGT  Rev: CACATCCTCAAAGAGCCTGA | 23 |
| ZSCAN4 | For: TGGAAATCAAGTGGCAAAAA  Rev: CTGCATGTGGACGTGGAC | 23 |
| CKM | For: ATGCCATTCGGTAACACCCAC  Rev: GCTTCTTGTAGAGTTCAAGGGTC | 100 |
| MYH8 | For: AATGCAAGTGCTATTCCAGAGG  Rev: ACAGACAGCTTGTGTTCTTGTT | 100 |
| MYOG | For: GGGGAAAACTACCTGCCTGTC  Rev: AGGCGCTCGATGTACTGGAT | 100 |
| RPL13A | For: AACCTCCTCCTTTTCCAAGC  Rev: GCAGTACCTGTTTAGCCACGA | 23 |
| DUX4.CA | For: TAGGGGAAGAGGTAGACGGC  Rev: GGTTCCGGGATTCC | 41 |
| GAPDH | For: TGCACCACCAACTGCTTAGC  Rev: GGCATGGACTGTGGTCATGAG | 41 |
