# Supplementary material for: Flavones provide resistance to DUX4-induced toxicity via an mTor-independent mechanism
Source: Cell Death Dis. 2023 Nov 16;14(11):749. doi: 10.1038/s41419-023-06257-2 (PMC10654915; doi:10.1038/s41419-023-06257-2)
Supplement: Supplementary file 1 — Supp. Figures [file 41419_2023_6257_MOESM1_ESM.pdf]

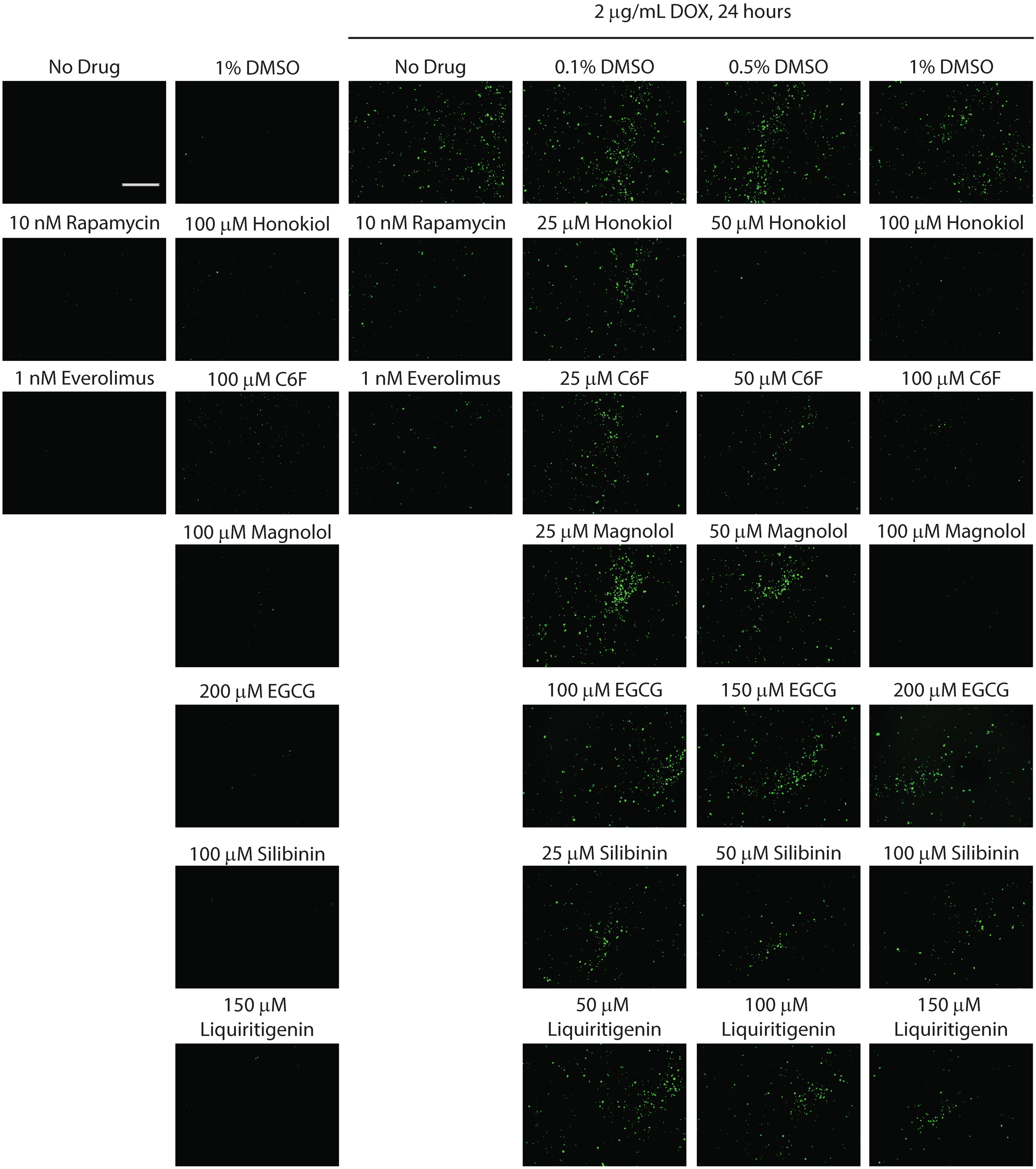
2 μg/mL DOX, 24 hours
No Drug
1% DMSO
No Drug
0.1% DMSO
0.5% DMSO
1% DMSO
10 nM Rapamycin
100 μM Honokiol
10 nM Rapamycin
25 μM Honokiol
50 μM Honokiol
100 μM Honokiol
1 nM Everolimus
100 μM C6F
1 nM Everolimus
25 μM C6F
50 μM C6F
100 μM C6F
100 μM Magnolol
25 μM Magnolol
50 μM Magnolol
100 μM Magnolol
200 μM EGCG
100 μM EGCG
150 μM EGCG
200 μM EGCG
100 μM Silibinin
25 μM Silibinin
50 μM Silibinin
100 μM Silibinin
150 μM Liquiritigenin
50 μM Liquiritigenin
100 μM Liquiritigenin
150 μM Liquiritigenin

Figure S1

A.

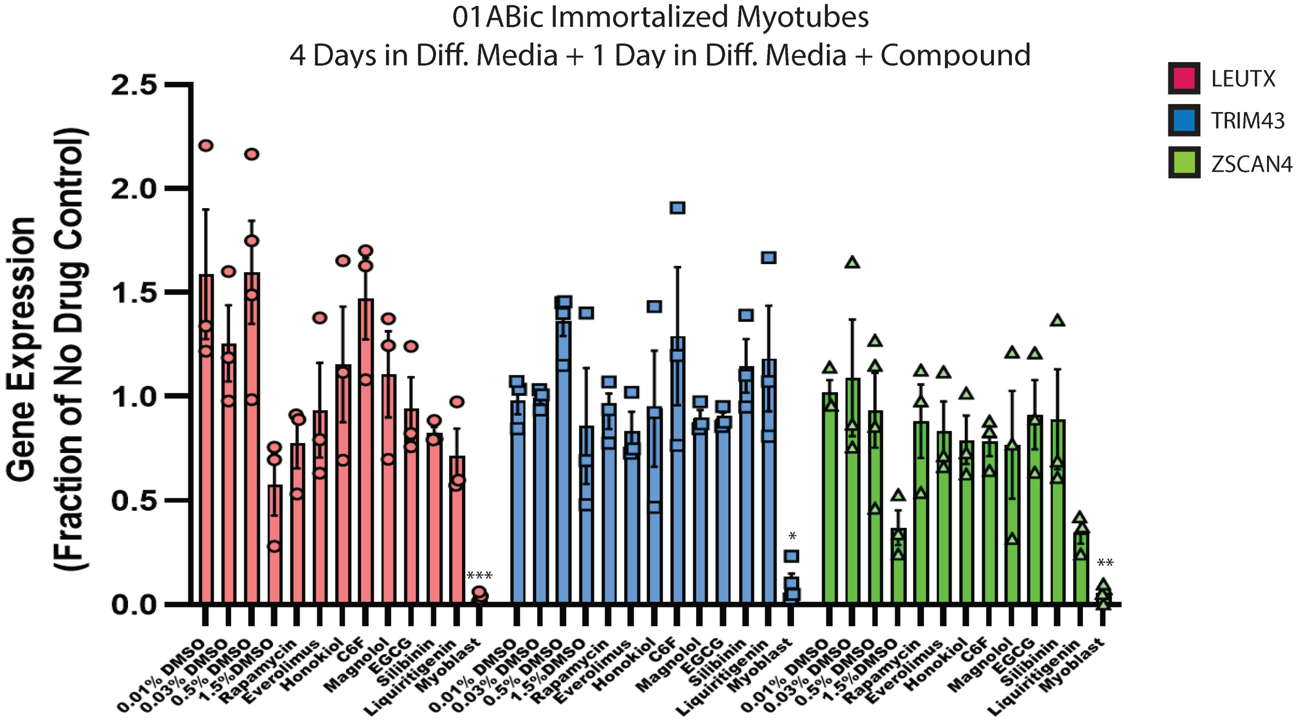

01ABic Immortalized Myotubes
4 Days in Diff. Media + 1 Day in Diff. Media + Compound
Gene Expression
(Fraction of No Drug Control)
2.5
2.0
1.5
1.0
0.5
0.0
LEUTX
TRIM43
ZSCAN4
***
*
**
0.01% DMSO
0.03% DMSO
0.5% DMSO
1.5%DMSO
Rapamycin
Everolimus
Honokiol
C6F
Magnolol
EGCG
Silibinin
Liquiritigenin
Myoblast

B.

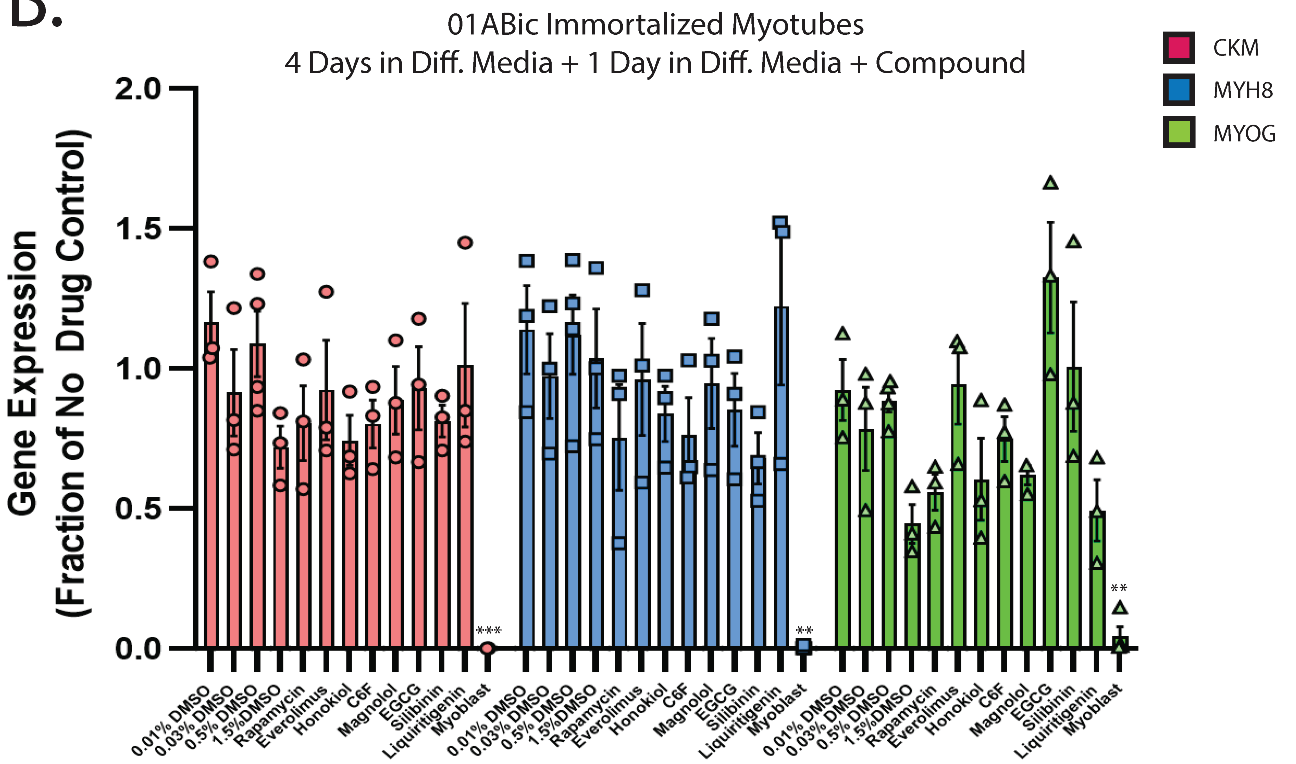

01ABic Immortalized Myotubes
4 Days in Diff. Media + 1 Day in Diff. Media + Compound
Gene Expression
(Fraction of No Drug Control)
2.0
1.5
1.0
0.5
0.0
CKM
MYH8
MYOG
***
**
**
0.01% DMSO
0.03% DMSO
0.5% DMSO
1.5%DMSO
Rapamycin
Everolimus
Honokiol
C6F
Magnolol
EGCG
Silibinin
Liquiritigenin
Myoblast

Figure S2

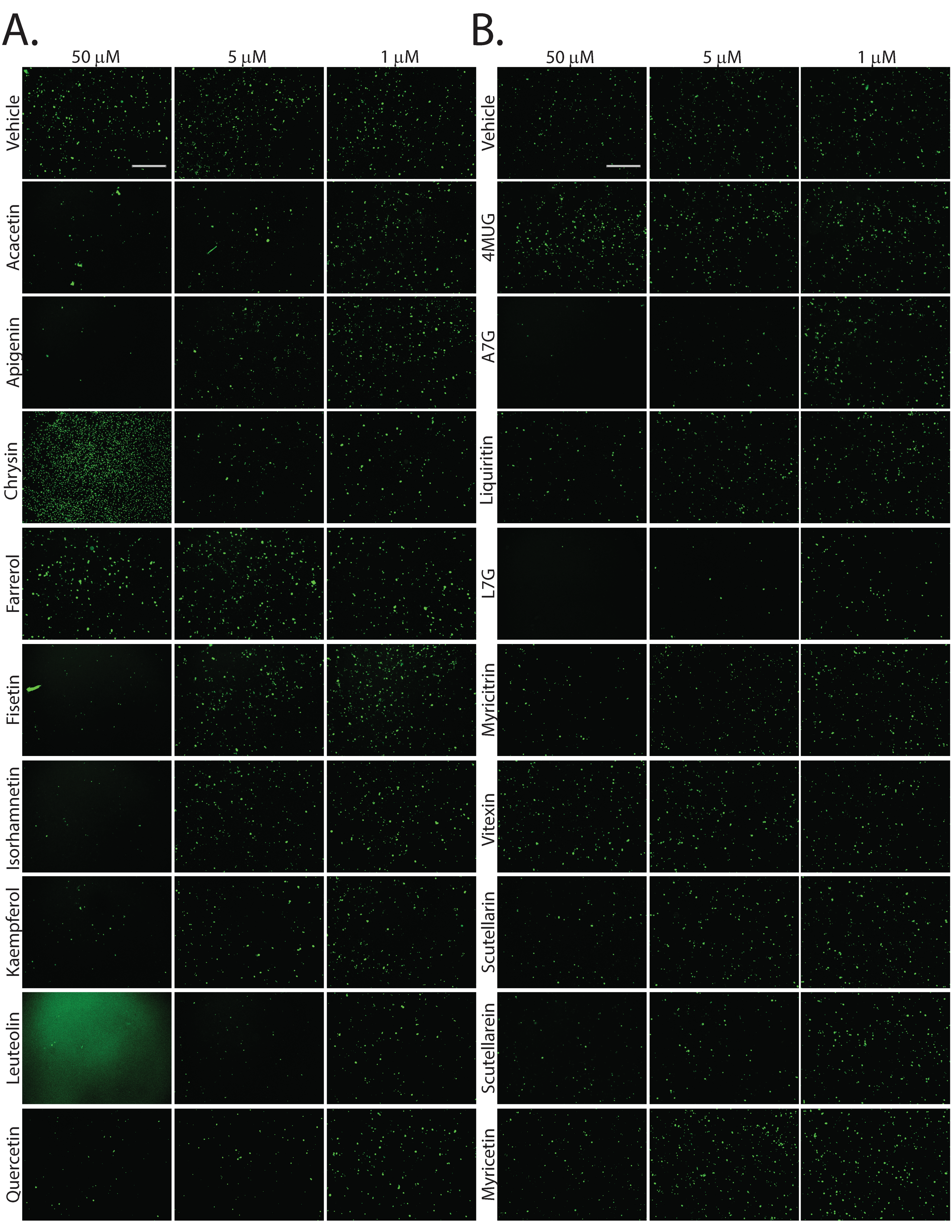
A.
50 μM
5 μM
1 μM
Vehicle
Acacetin
Apigenin
Chrysin
Farrerol
Fisetin
Isorhamnetin
Kaempferol
Leuteolin
Quercetin
B.
50 μM
5 μM
1 μM
Vehicle
4MUG
A7G
Liquiritin
L7G
Myricitrin
Vitexin
Scutellarin
Scutellarein
Myricetin

C.

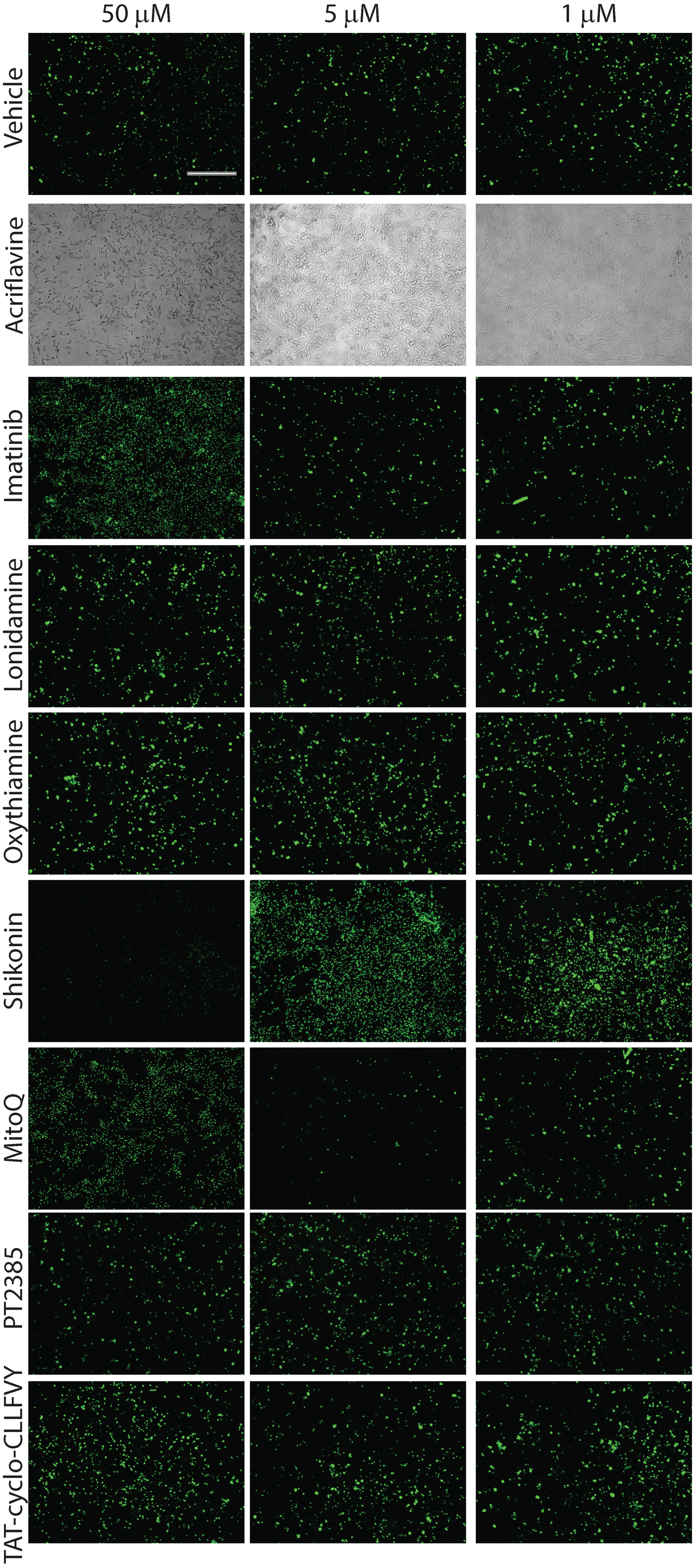
50 μM
5 μM
1 μM
Vehicle
Acriflavine
Imatinib
Lonidamine
Oxythiamine
Shikonin
MitoQ
PT2385
TAT-cyclo-CLLFVY

Figure S3

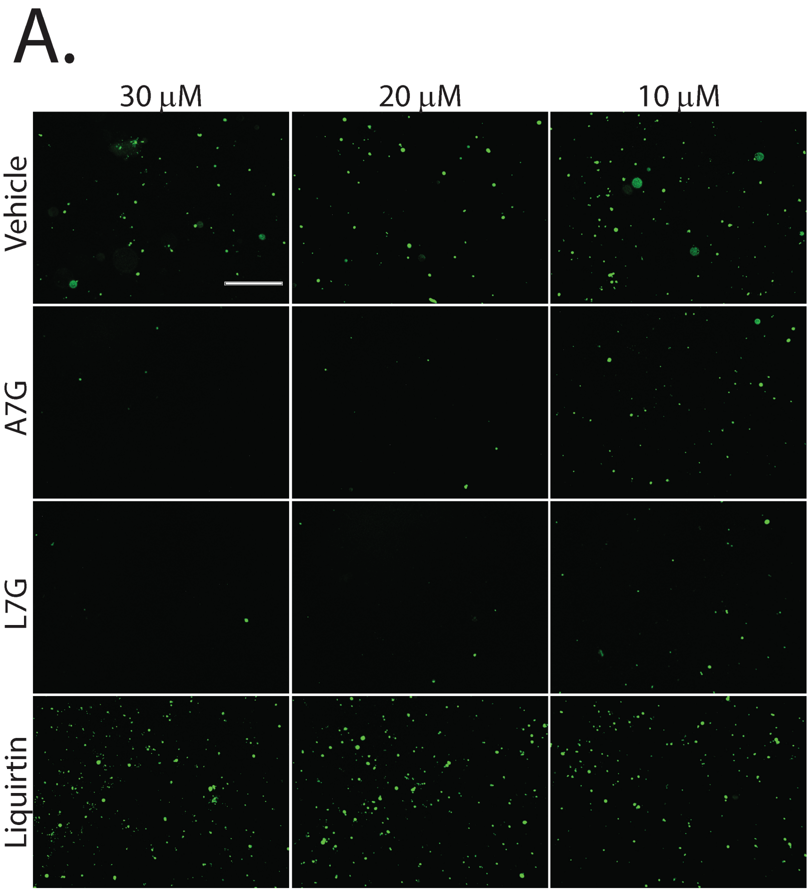
A.
30 μM
20 μM
10 μM
Vehicle
A7G
L7G
Liquirtin

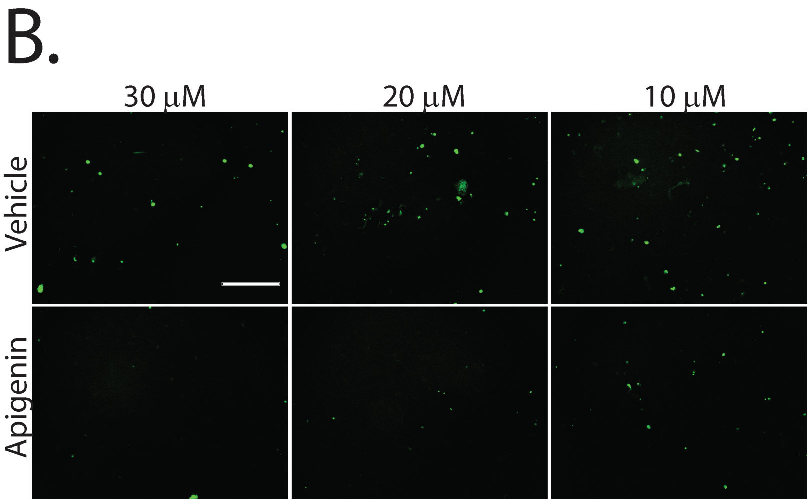
B.
30 μM
20 μM
10 μM
Vehicle
Apigenin

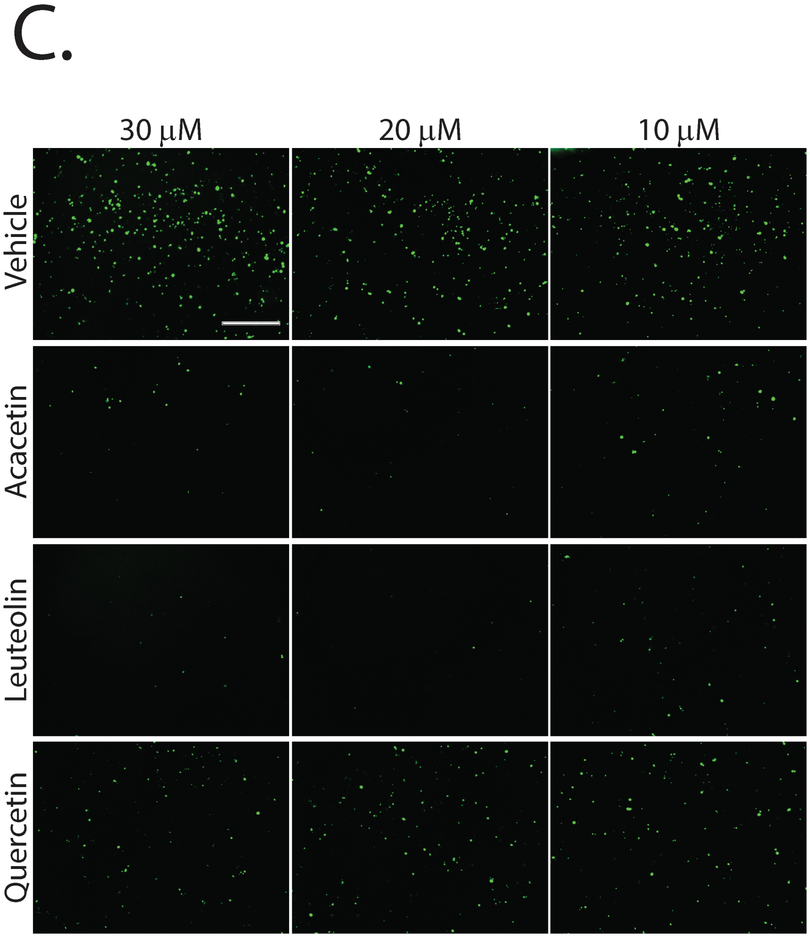
C.
30 μM
20 μM
10 μM
Vehicle
Acacetin
Leuteolin
Quercetin

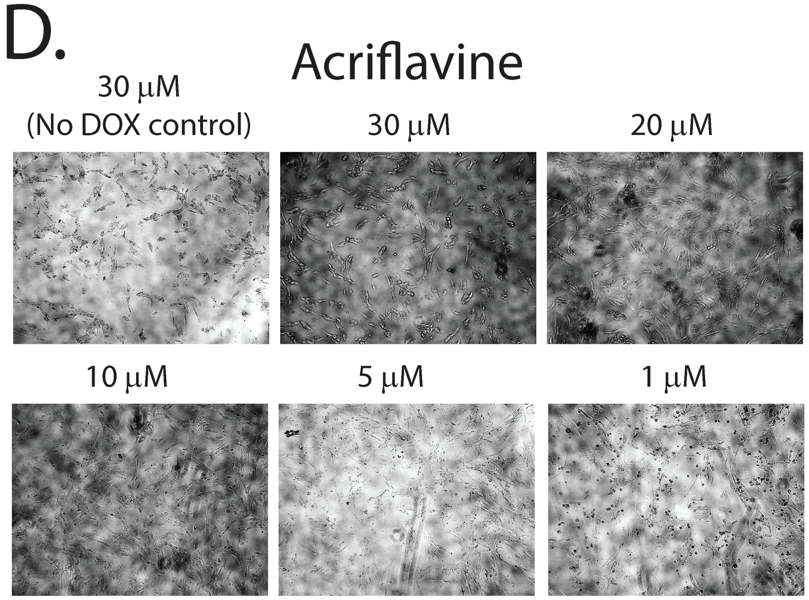
D.
Acriflavine
30 μM
(No DOX control)
30 μM
20 μM
10 μM
5 μM
1 μM

Figure S4

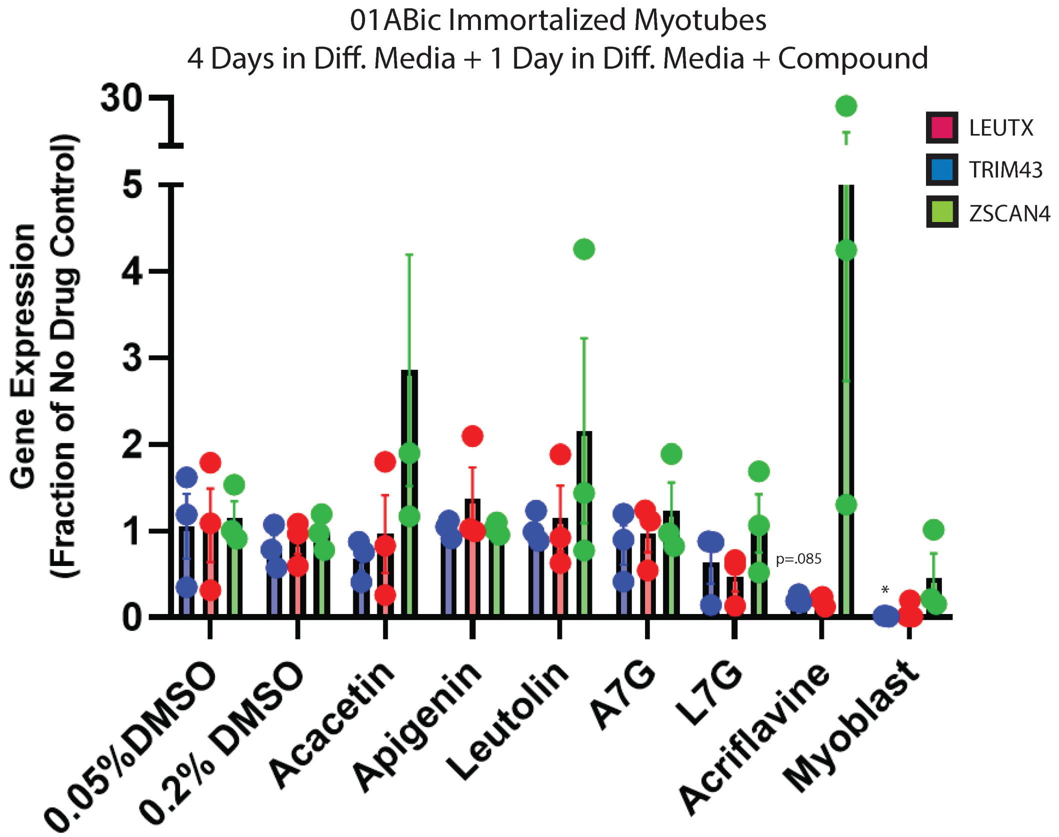
01ABic Immortalized Myotubes
4 Days in Diff. Media + 1 Day in Diff. Media + Compound
Gene Expression
(Fraction of No Drug Control)
30
5
4
3
2
1
0
LEUTX
TRIM43
ZSCAN4
p=.085
*
0.05%DMSO
0.2% DMSO
Acacetin
Apigenin
Leutolin
A7G
L7G
Acriflavine
Myoblast

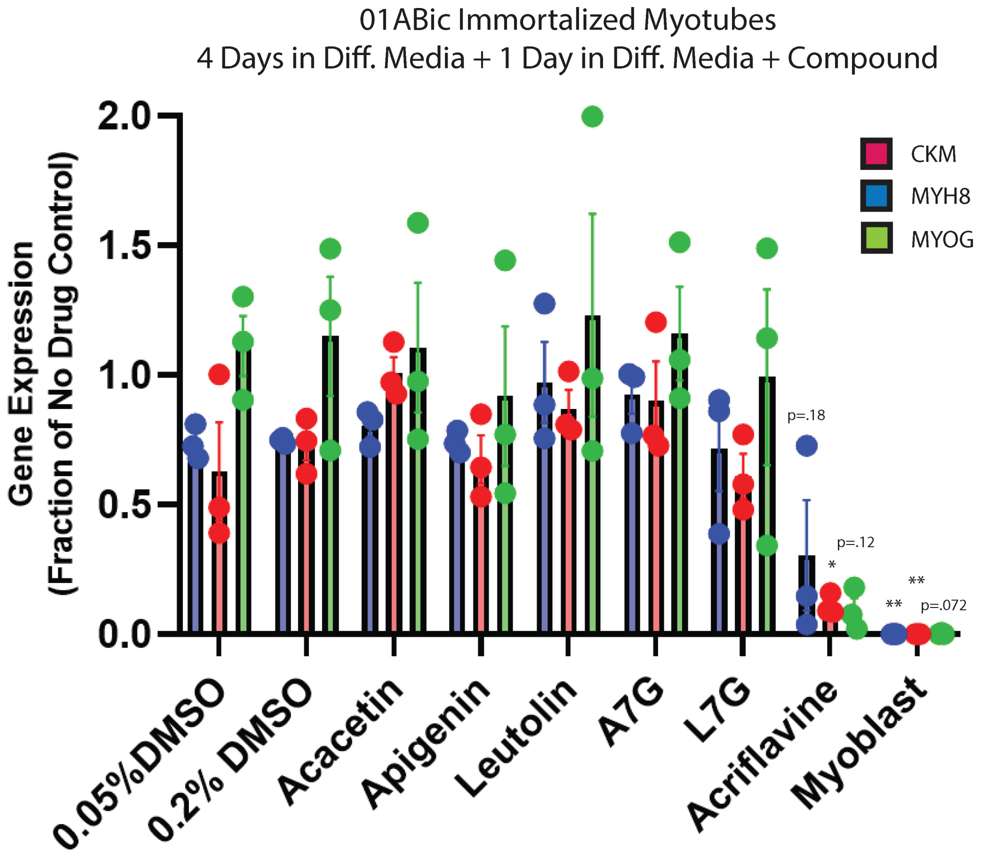
01ABic Immortalized Myotubes
4 Days in Diff. Media + 1 Day in Diff. Media + Compound
Gene Expression
(Fraction of No Drug Control)
2.0
1.5
1.0
0.5
0.0
CKM
MYH8
MYOG
p=.18
p=.12
*
**
**
p=.072
0.05%DMSO
0.2% DMSO
Acacetin
Apigenin
Leutolin
A7G
L7G
Acriflavine
Myoblast

Figure S5

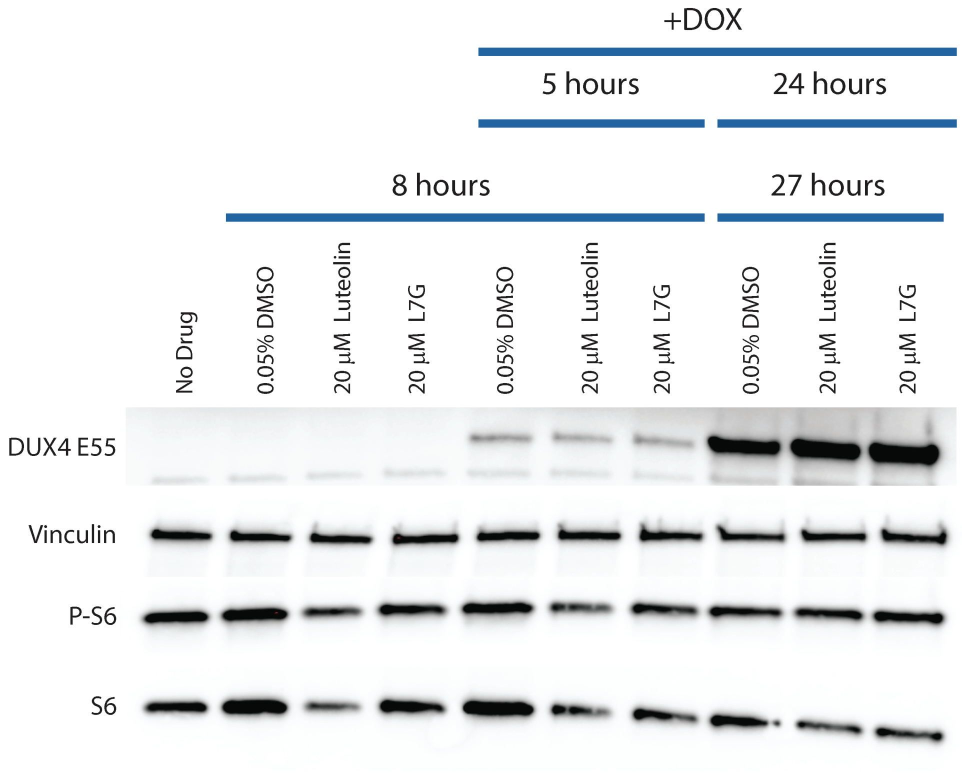
+DOX
5 hours
24 hours
8 hours
27 hours
No Drug
0.05% DMSO
20 µM Luteolin
20 µM L7G
0.05% DMSO
20 µM Luteolin
20 µM L7G
0.05% DMSO
20 µM Luteolin
20 µM L7G
DUX4 E55
Vinculin
P-S6
S6

Figure S6

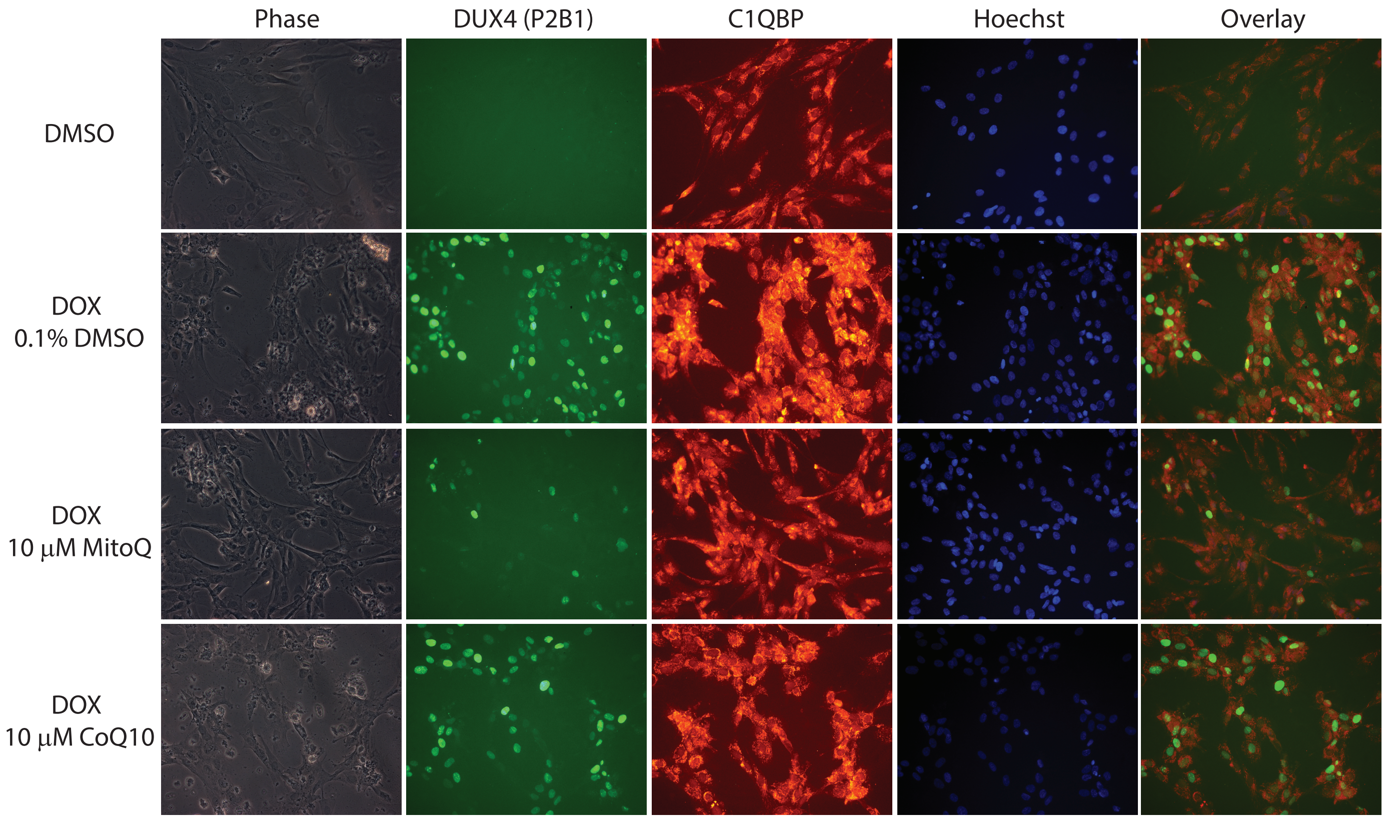
Phase
DUX4 (P2B1)
C1QBP
Hoechst
Overlay
DMSO
DOX
0.1% DMSO
DOX
10 µM MitoQ
DOX
10 µM CoQ10

Figure S7

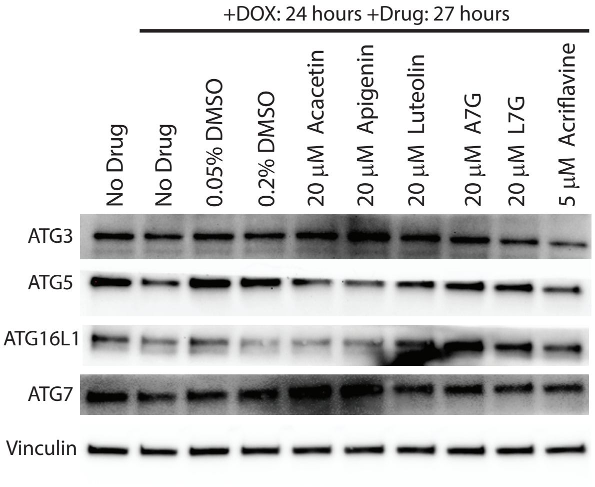

+DOX: 24 hours +Drug: 27 hours
No Drug
No Drug
0.05% DMSO
0.2% DMSO
20 µM Acacetin
20 µM Apigenin
20 µM Luteolin
20 µM A7G
20 µM L7G
5 µM Acriflavine
ATG3
ATG5
ATG16L1
ATG7
Vinculin

Figure S8

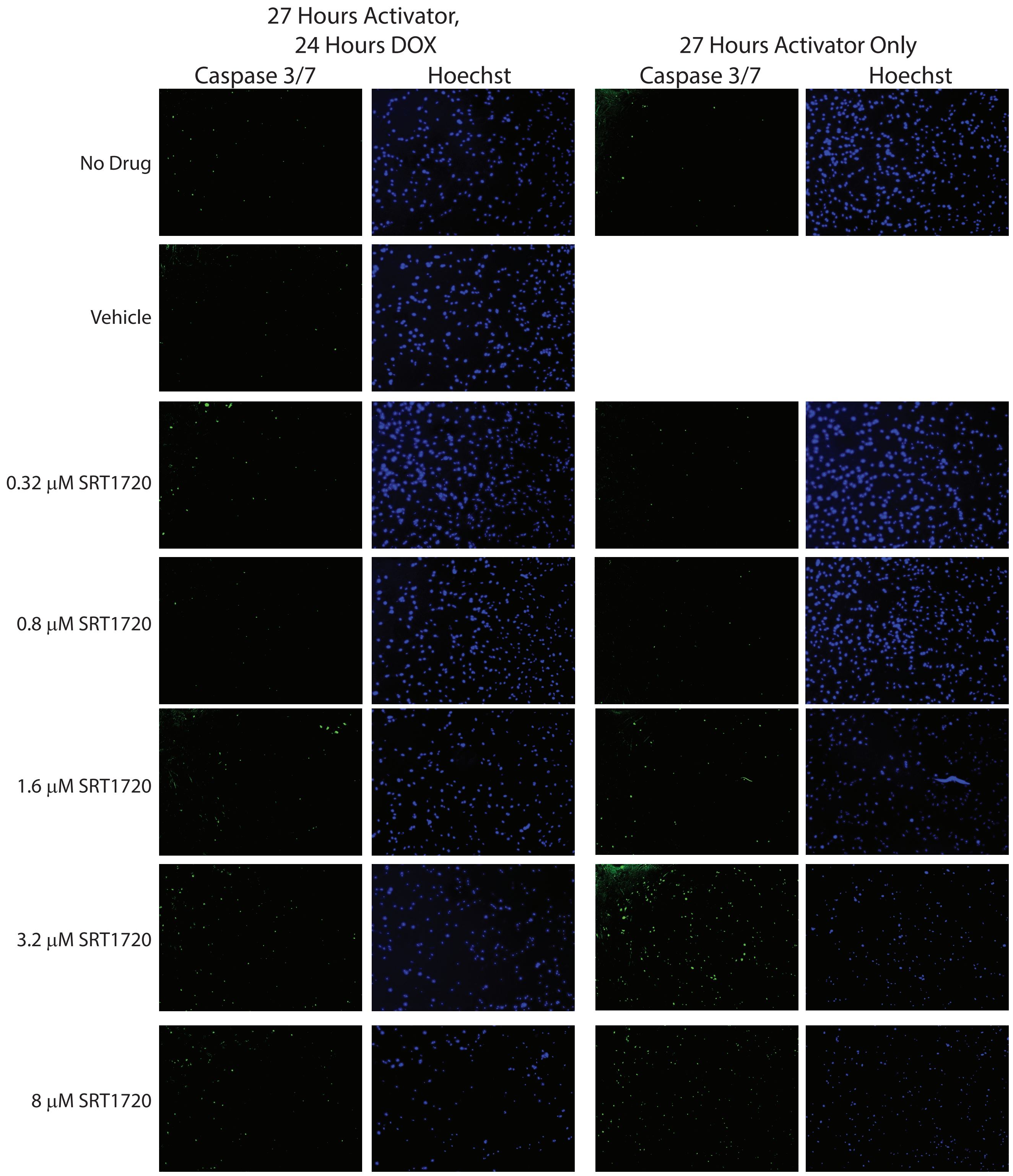
27 Hours Activator,
24 Hours DOX
27 Hours Activator Only
Caspase 3/7
Hoechst
Caspase 3/7
Hoechst
No Drug
Vehicle
0.32 μM SRT1720
0.8 μM SRT1720
1.6 μM SRT1720
3.2 μM SRT1720
8 μM SRT1720

Figure S9

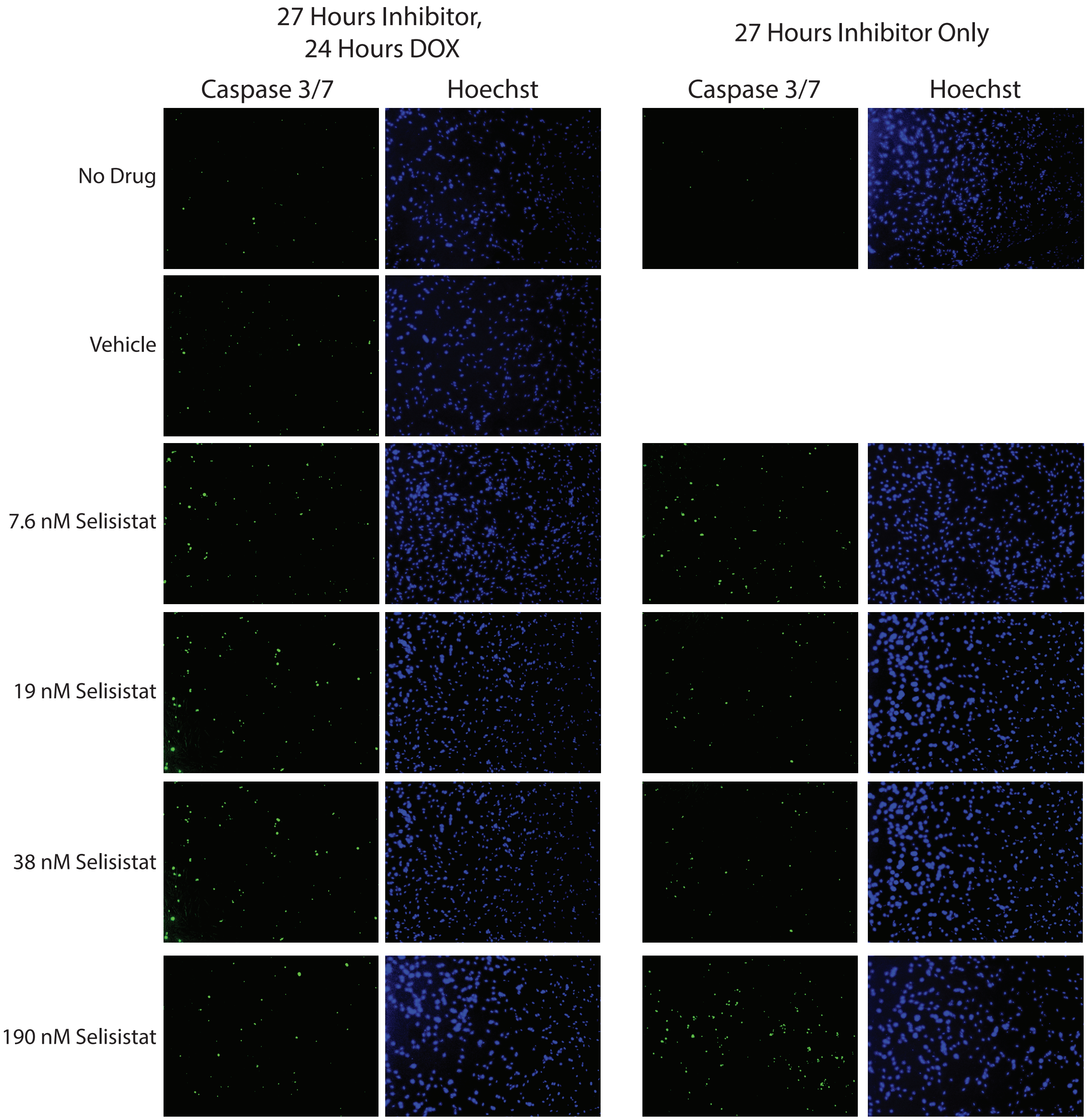
27 Hours Inhibitor,
24 Hours DOX
27 Hours Inhibitor Only
Caspase 3/7
Hoechst
Caspase 3/7
Hoechst
No Drug
Vehicle
7.6 nM Selisistat
19 nM Selisistat
38 nM Selisistat
190 nM Selisistat

Figure S10

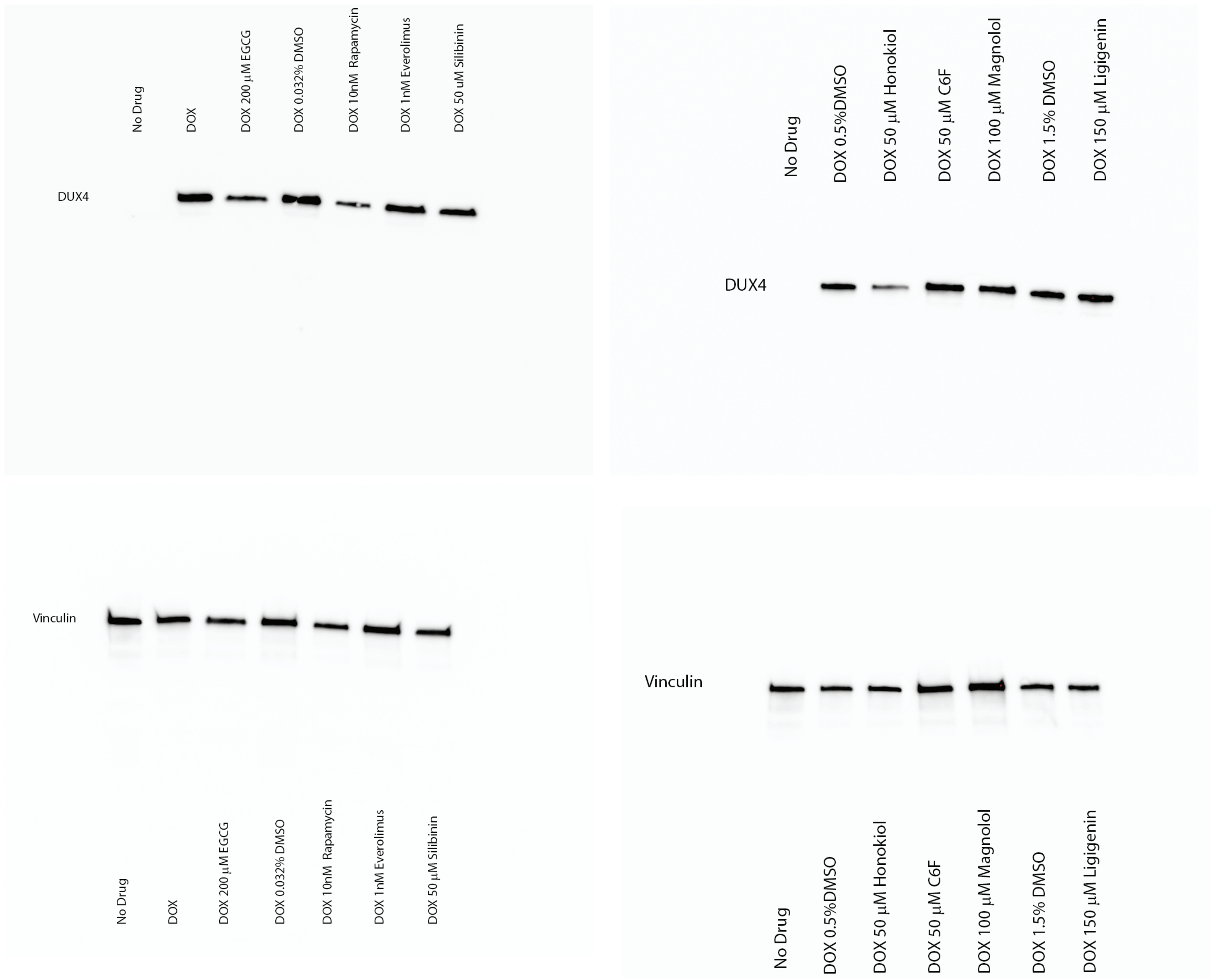

Figure S11: Uncropped western blots presented in Figure 1E

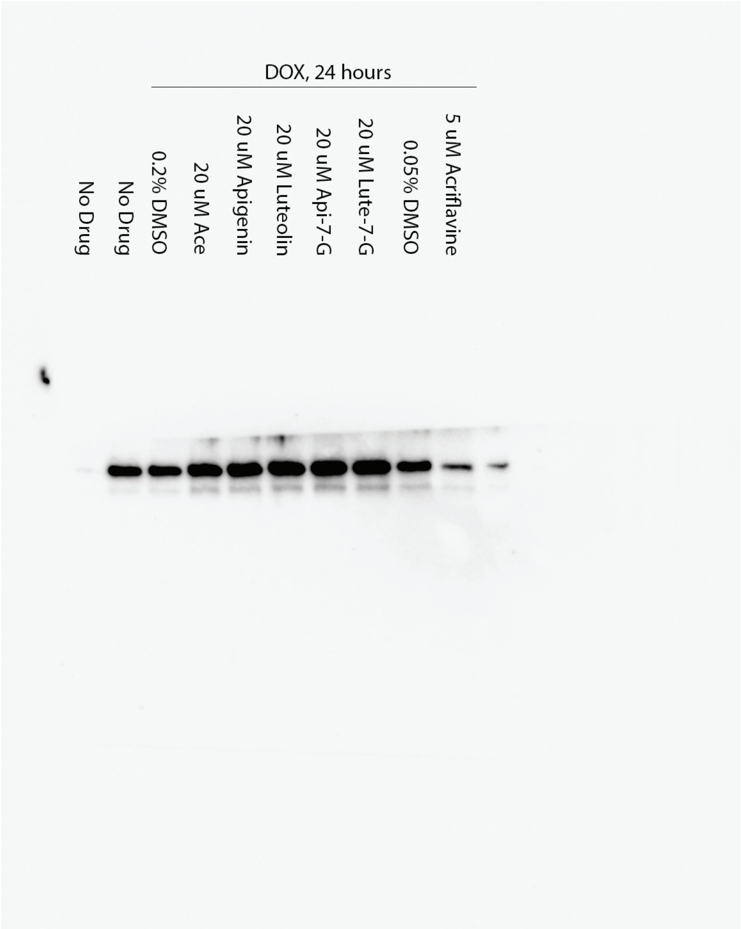

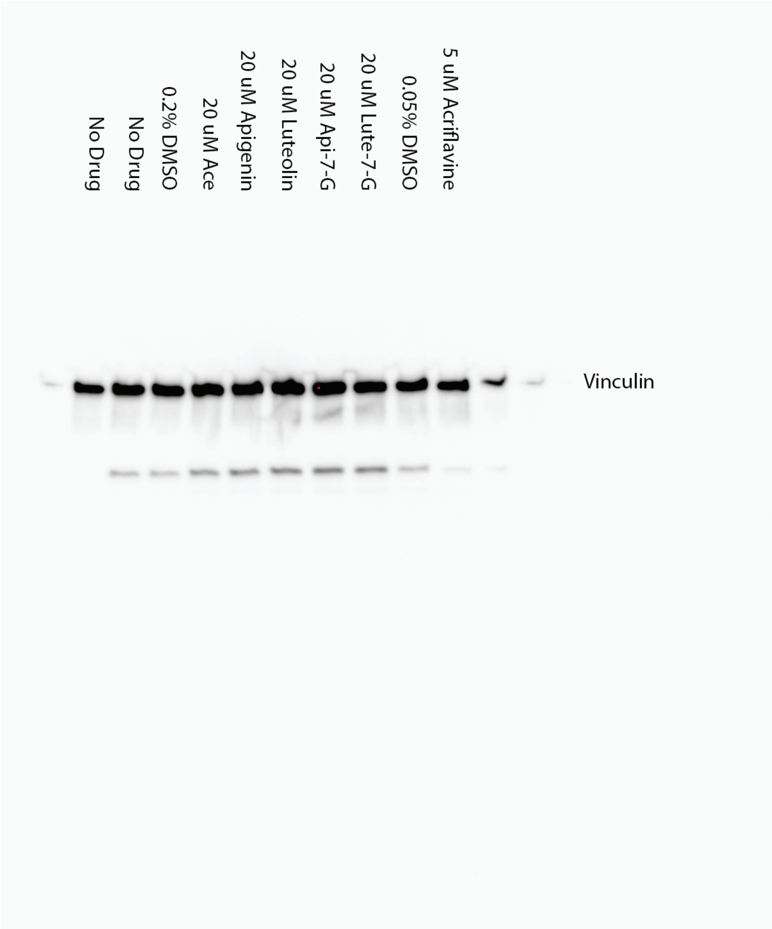

Figure S12: Uncropped western blots presented in Figure 3E

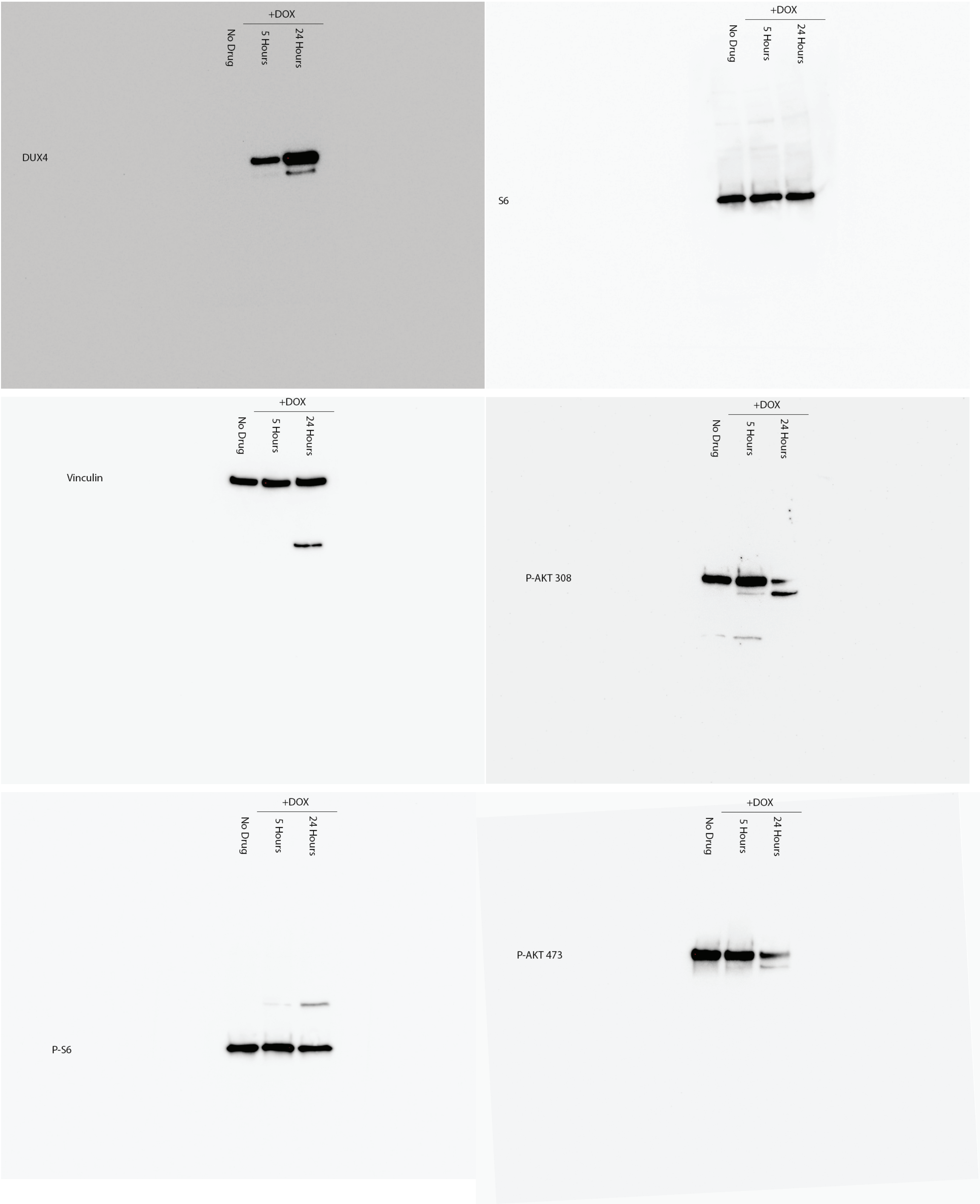

Figure S13: Uncropped western blots presented in Figure 5A

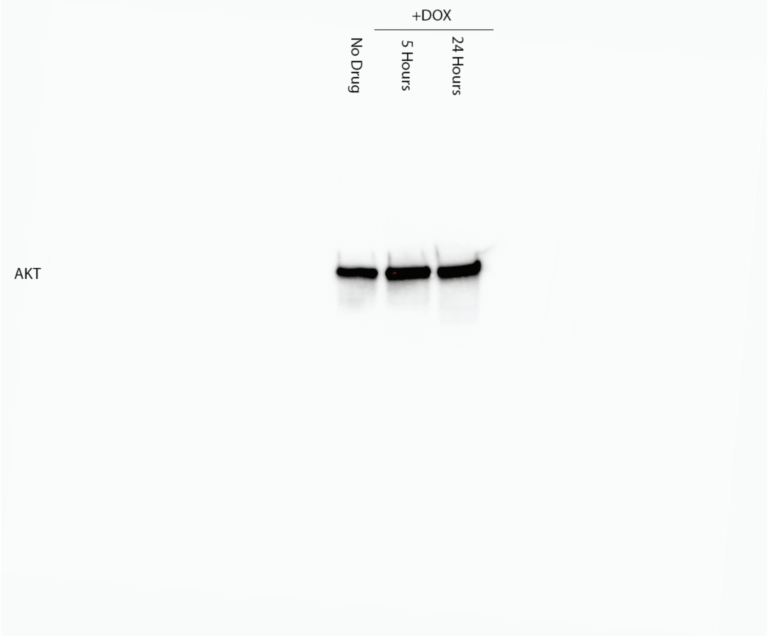

Figure S13: Uncropped western blots presented in Figure 5A continued

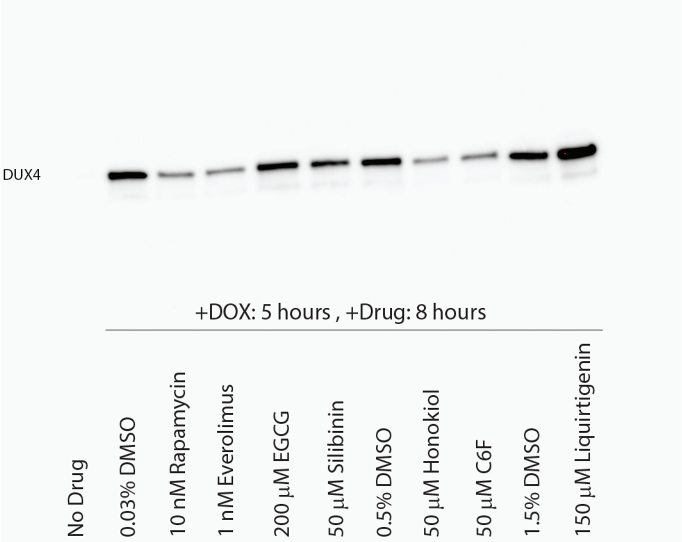

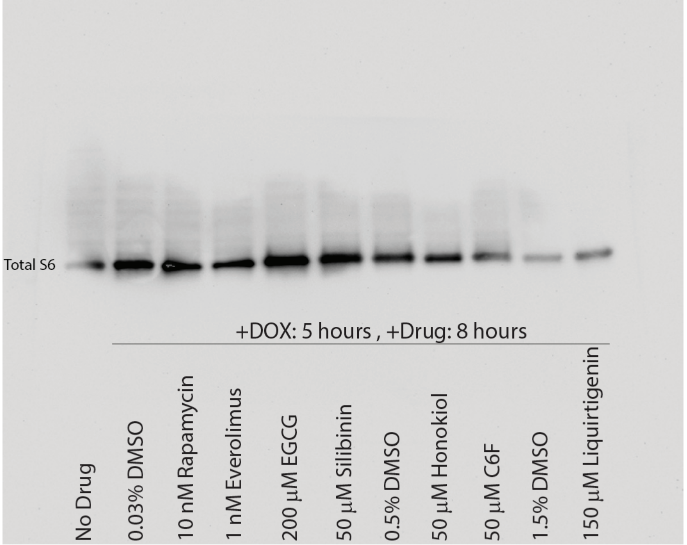

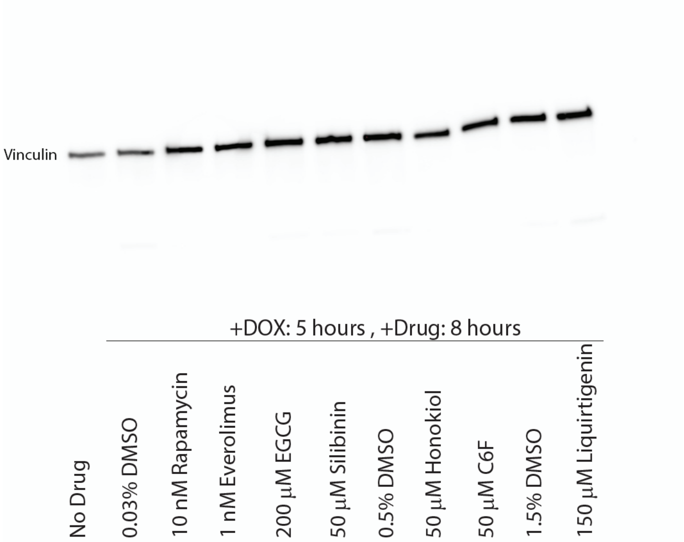

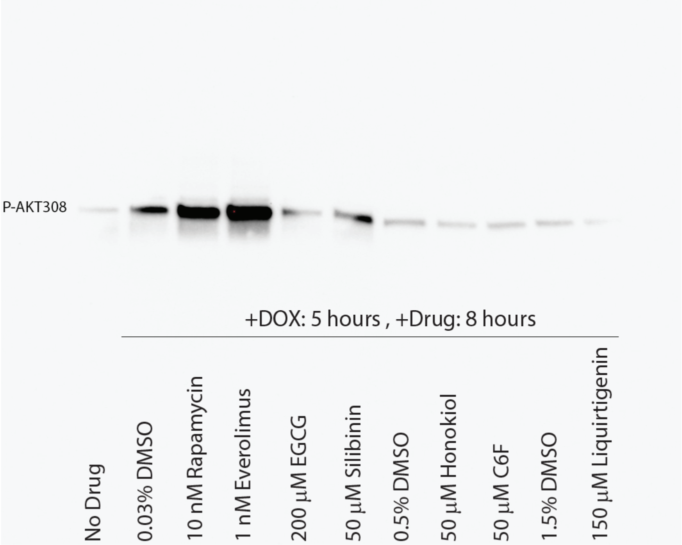

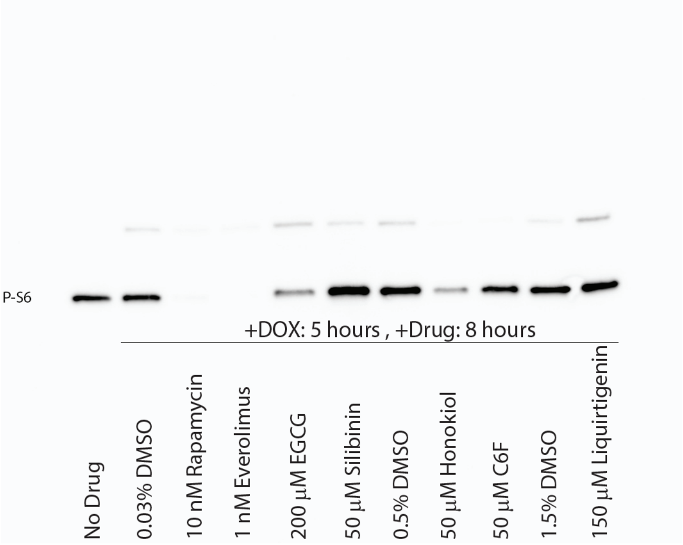

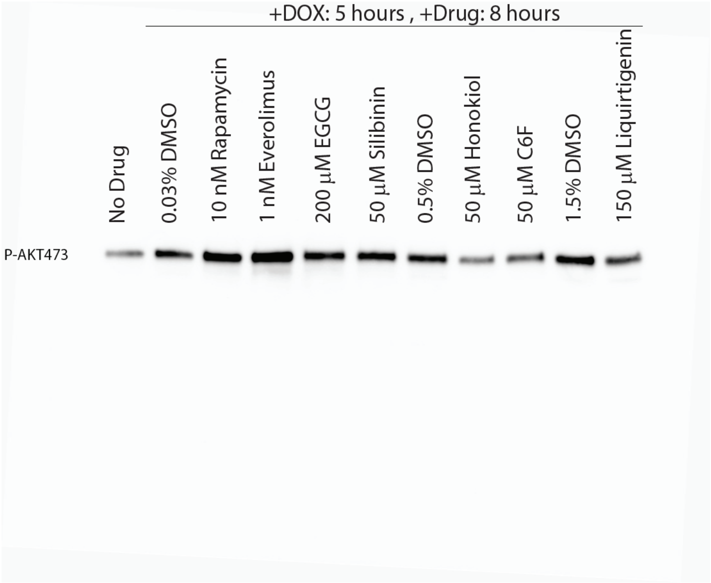

Figure S14: Uncropped western blots presented in Figure 5B

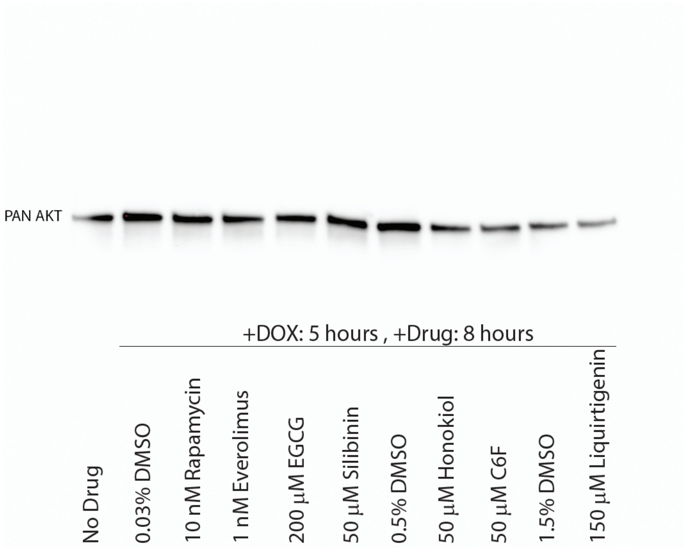

Figure S14: Uncropped western blots presented in Figure 5B continued

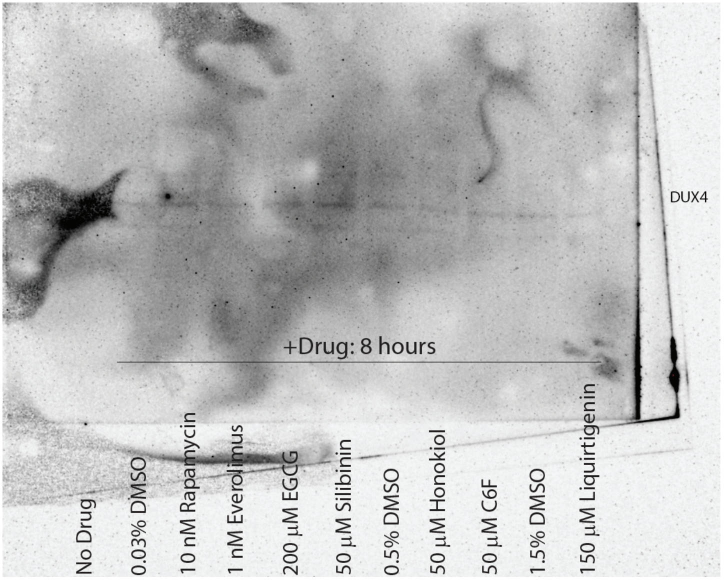

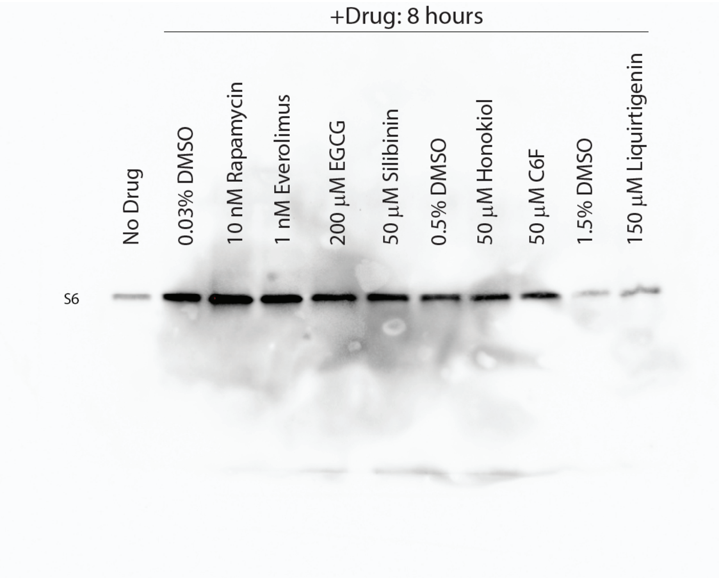

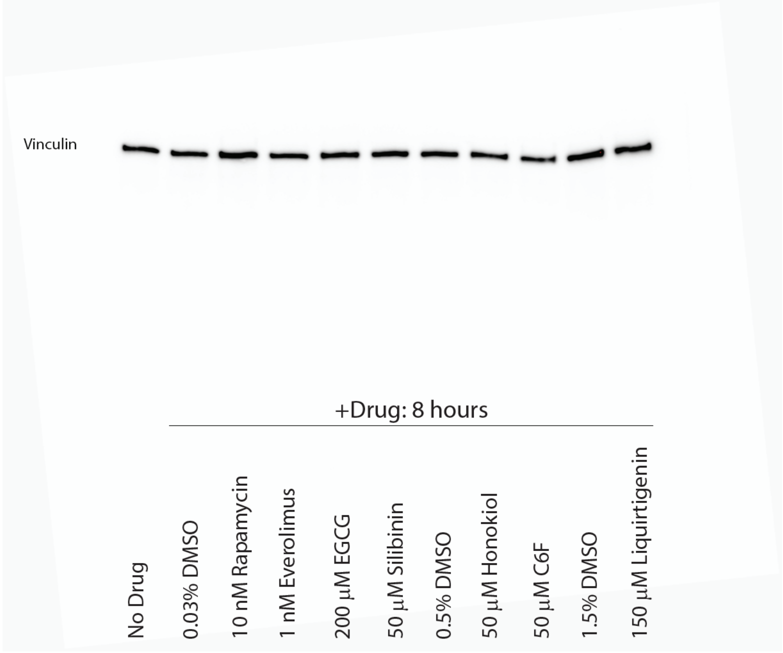

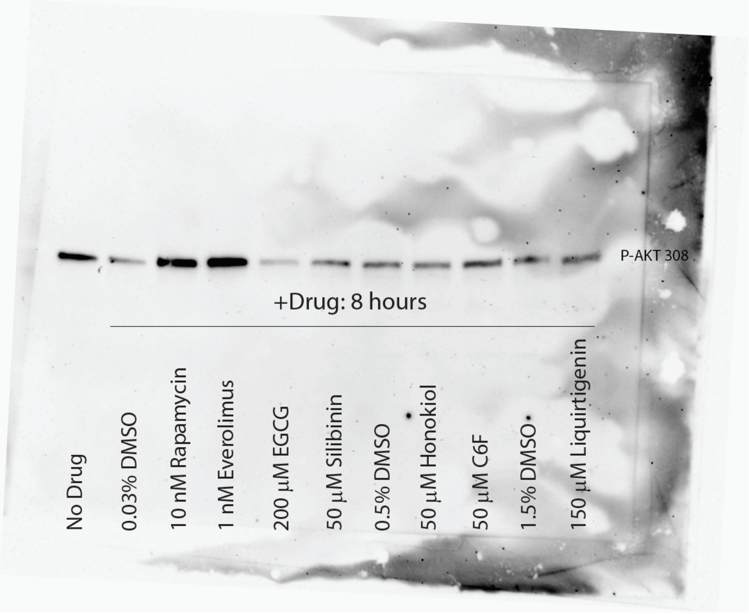

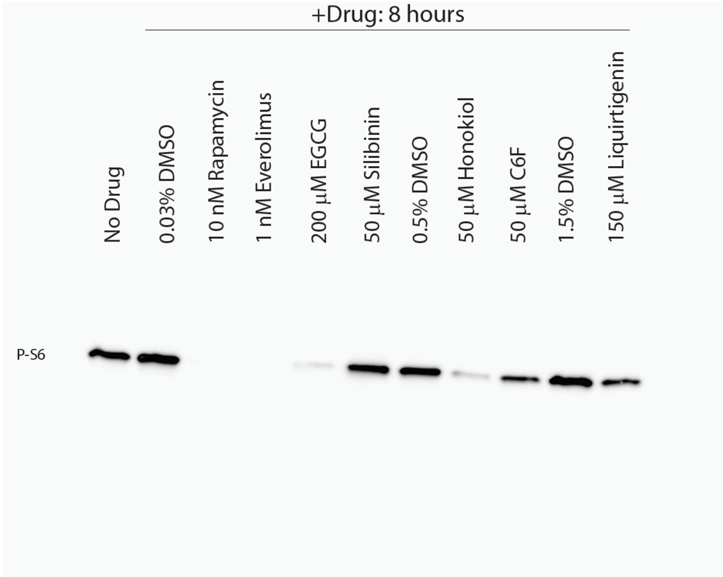

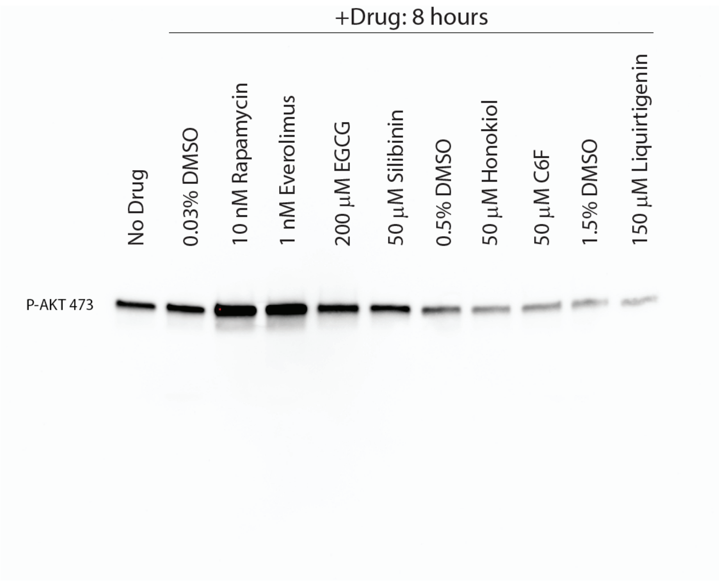

Figure S15: Uncropped western blots presented in Figure 5C

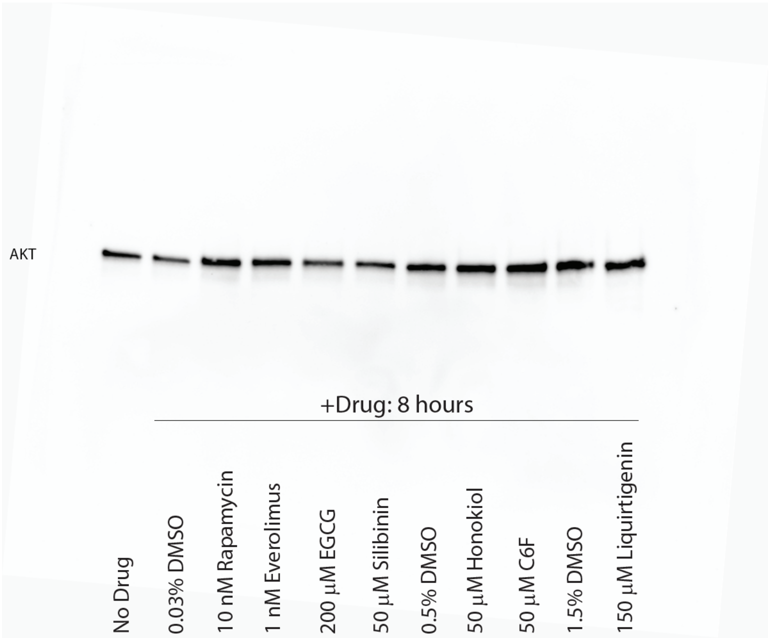

Figure S15: Uncropped western blots presented in Figure 5C Continued

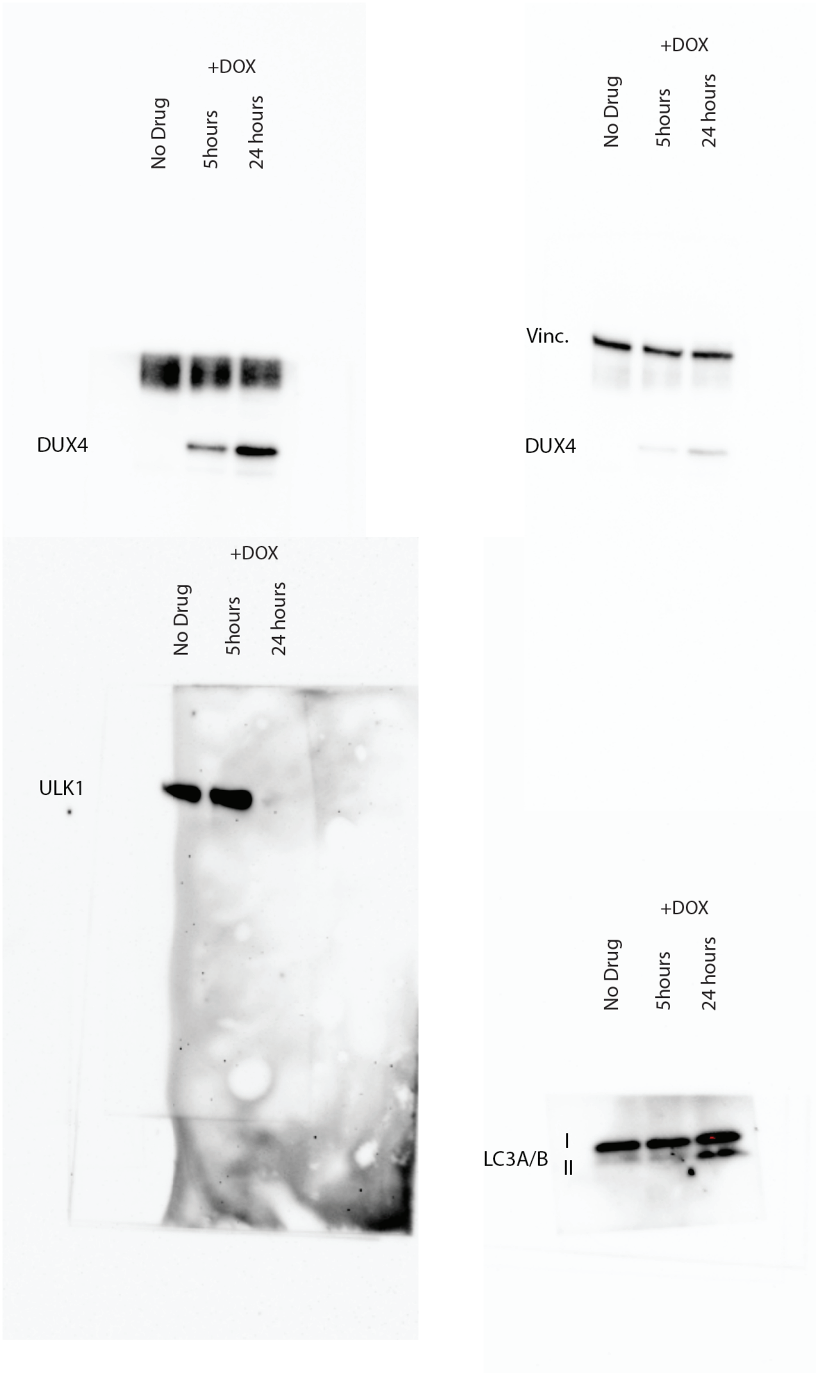

Figure S16: Uncropped western blots presented in Figure 6A

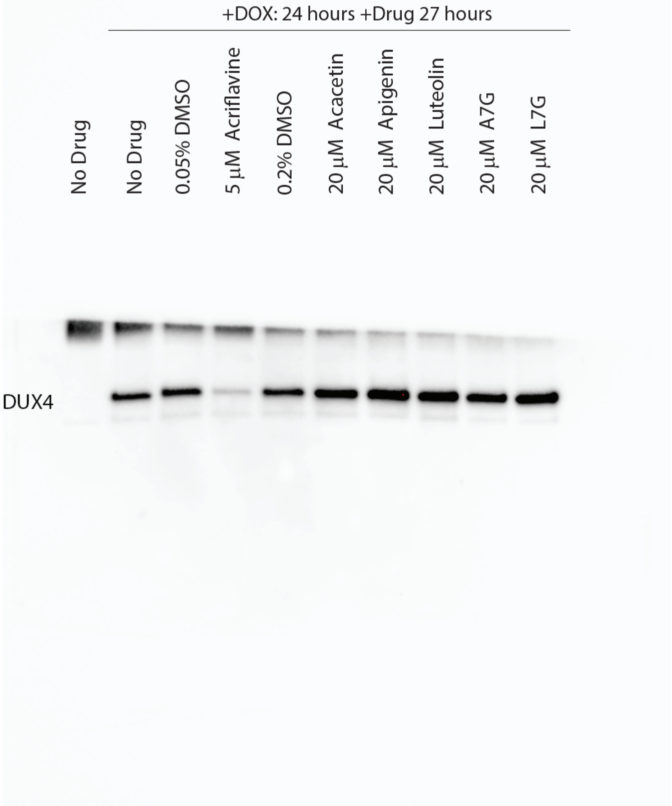

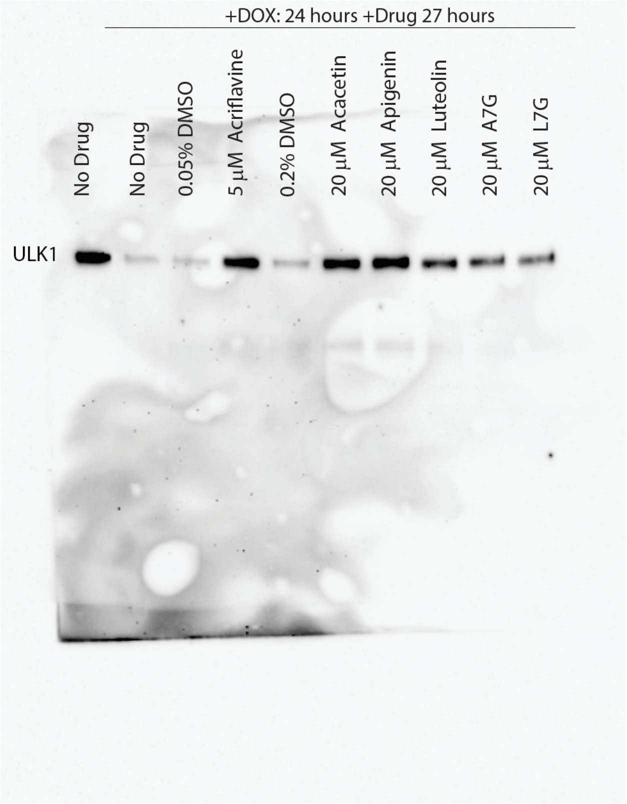

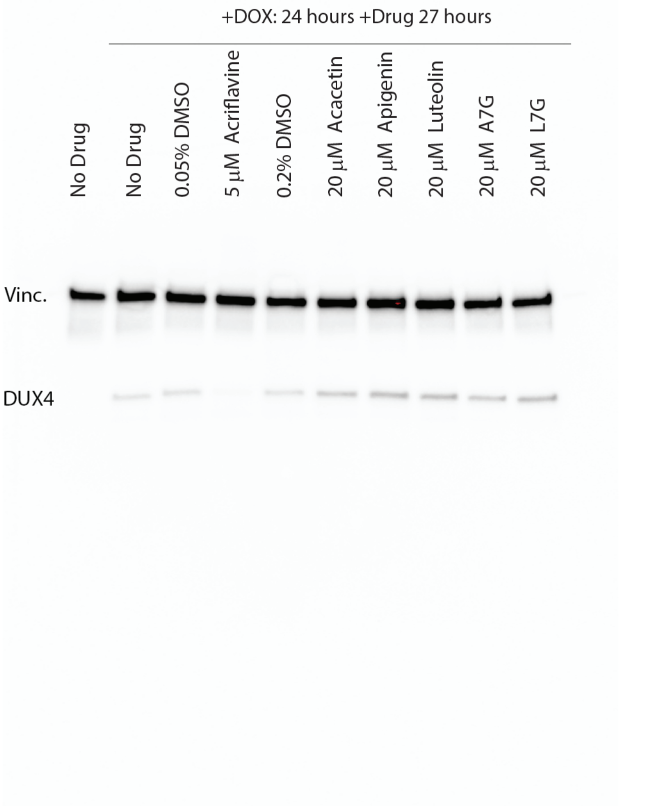

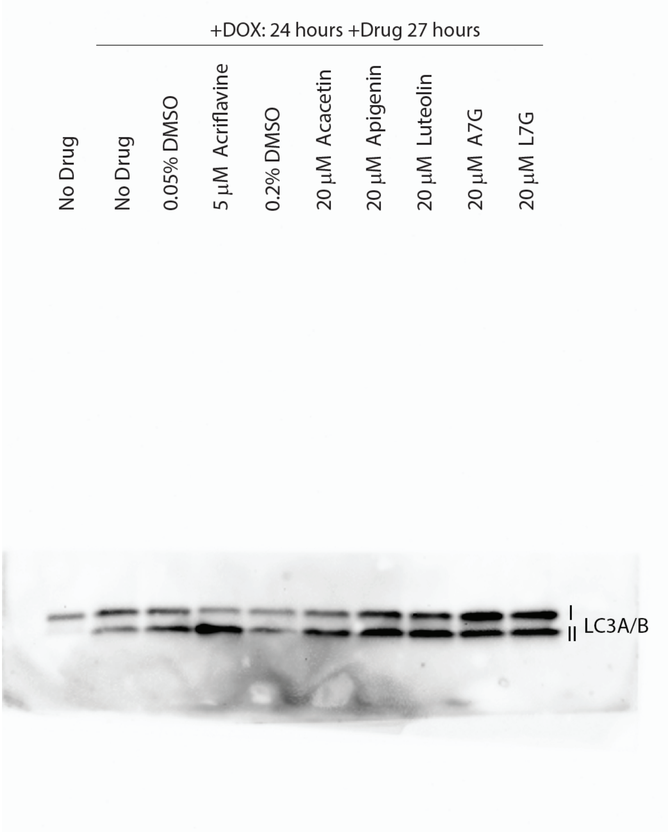

Figure S17: Uncropped western blots presented in Figure 6B

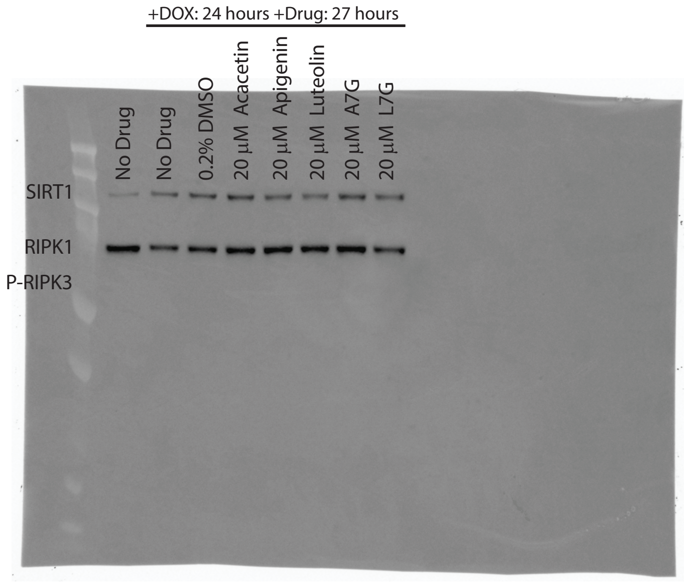

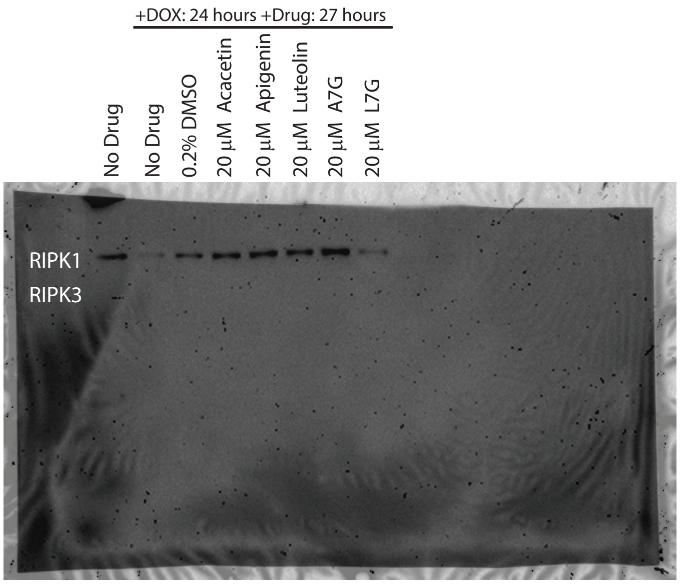

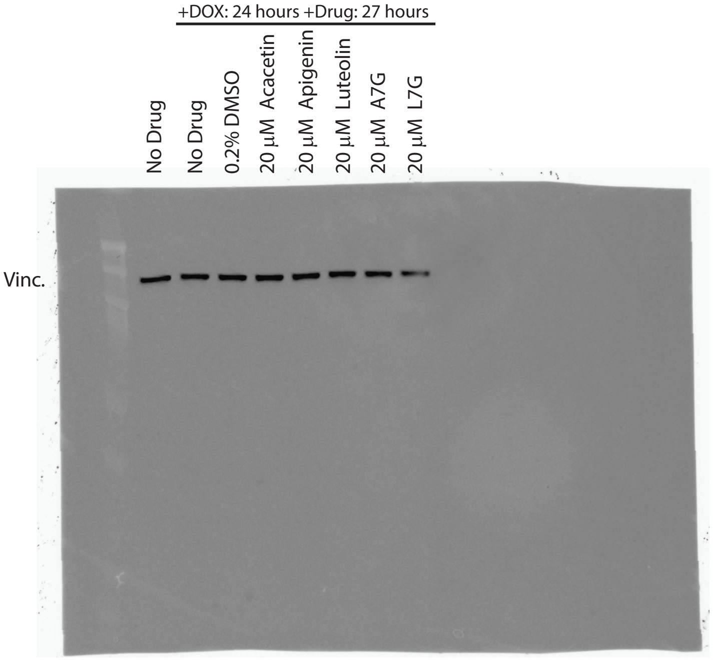

Figure S18: Uncropped western blots presented in Figure 6C

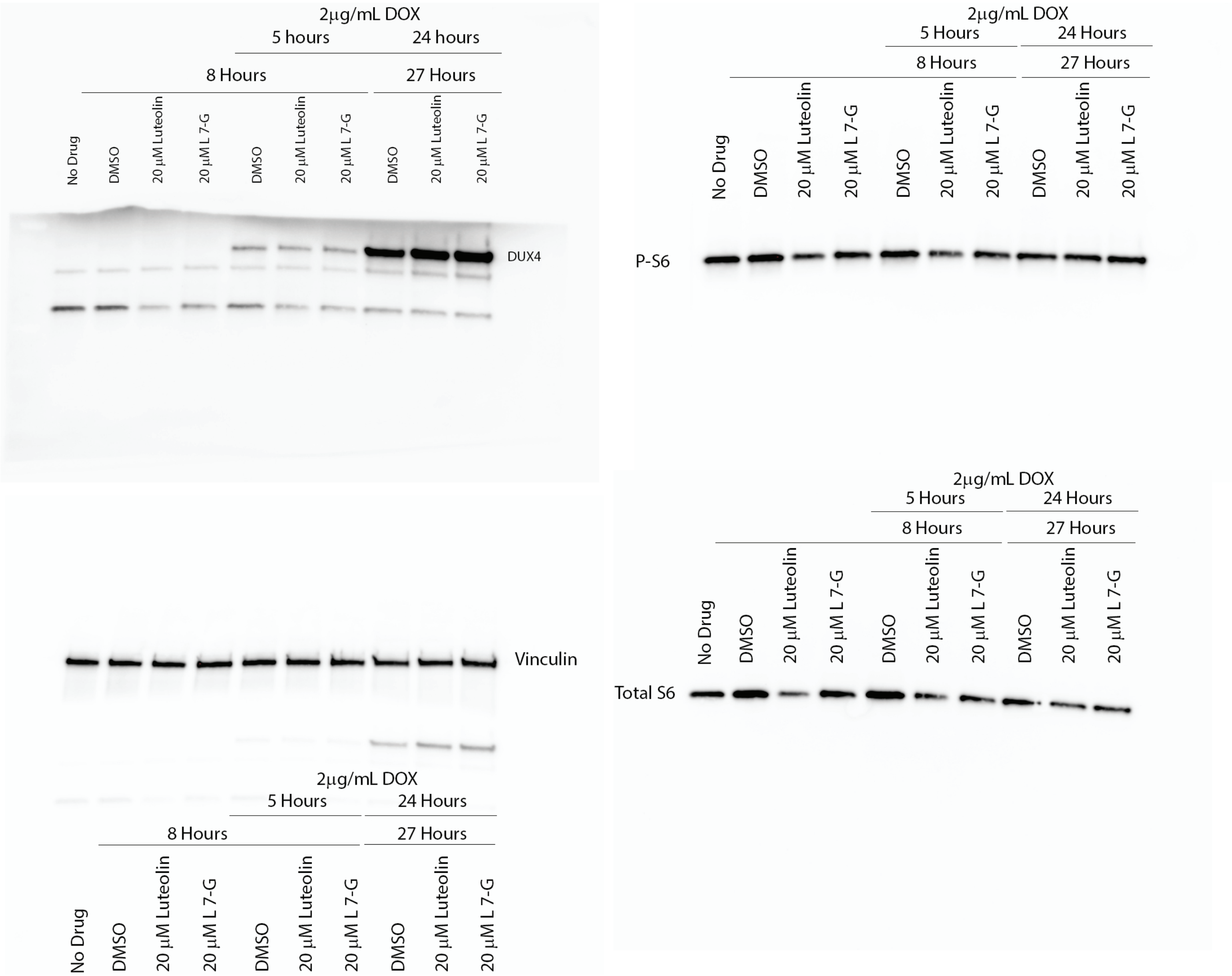

Figure S19: Uncropped western blots presented in Figure S6

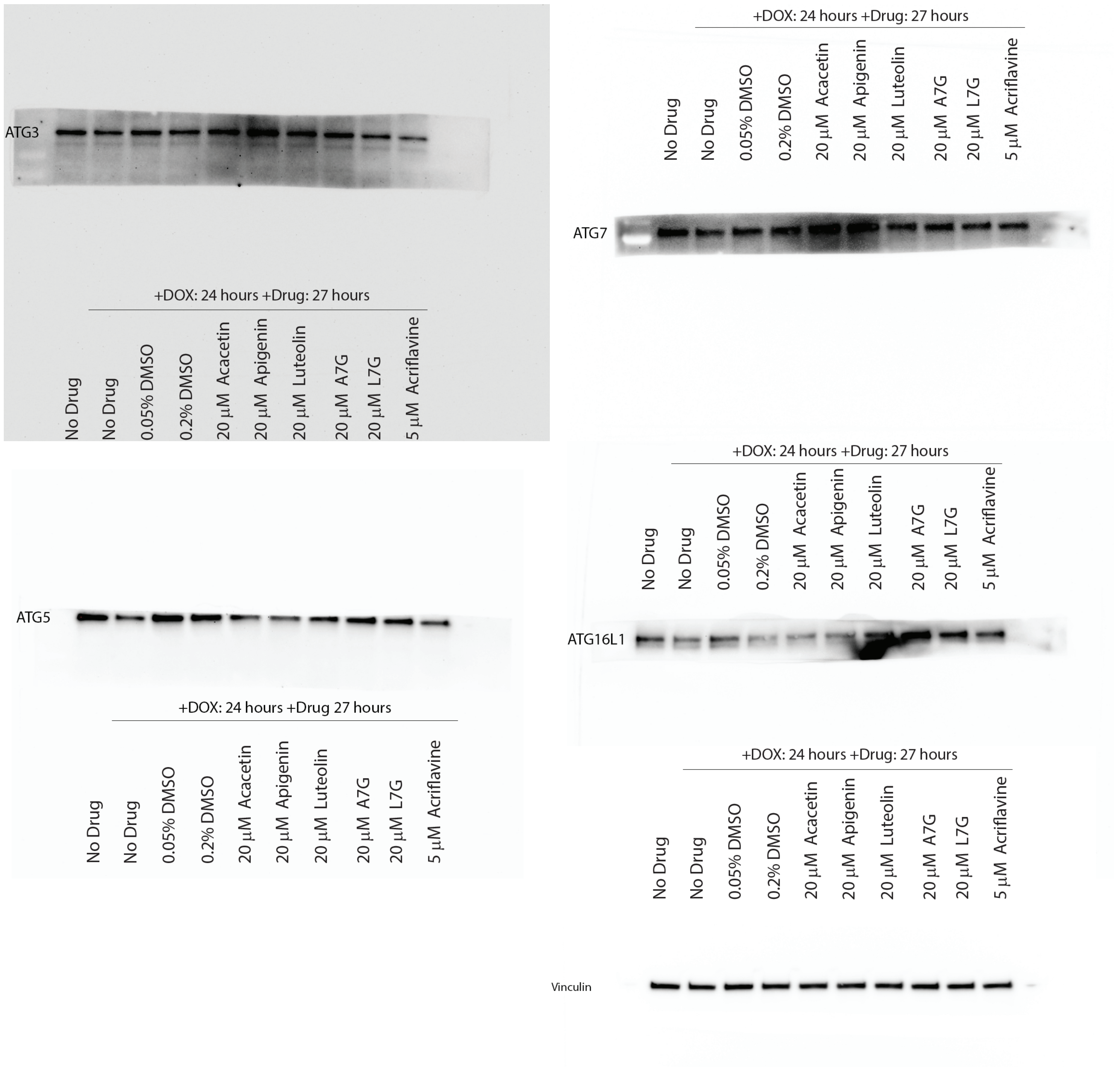

Figure S20: Uncropped western blots presented in Figure S8
